# Immunological Evaluation of Goats Immunized with a Commercial Vaccine against Johne’s Disease

**DOI:** 10.3390/vaccines10040518

**Published:** 2022-03-26

**Authors:** John P. Bannantine, Judith R. Stabel, Vivek Kapur

**Affiliations:** 1 USDA-Agricultural Research Service, National Animal Disease Center, Ames, IA 50010, USA; judy.stabel@usda.gov; 2 Department of Animal Science, Huck Institutes of the Life Sciences, The Pennsylvania State University, University Park, PA 16802, USA; vkapur@psu.edu

**Keywords:** Johne’s disease, goats, vaccine, cytokines, immune response, *Mycobacterium avium*

## Abstract

Johne’s disease affects ruminants causing an economic burden to dairy, meat and wool industries. Vaccination against *Mycobacterium avium* subspecies *paratuberculosis* (*Map*), which causes Johne’s disease, is a primary intervention for disease control in livestock. Previously, a comprehensive, multi-institutional vaccine trial for Johne’s disease was conducted to test the efficacy of live attenuated *Map* strains. Here, we report the humoral and cell-mediated immune responses from kid goats enrolled in that trial. Both vaccinated and unvaccinated animals showed IFN-γ stimulation and proliferation of T cell subpopulations on challenge with *Map*. CD4+, CD25+ and γδ cells from cultured PBMCs in the vaccinated goats showed significantly greater proliferation responses on stimulation with *Map* antigens. The increase in CD44+ and decrease in CD62L+ cells suggest that vaccine administration reduced the inflammatory responses associated with *Map* infection. Overall, a stronger antibody response was observed in the infected goats as compared to vaccinated goats. Two independent experimental approaches were used to identify differences in the antibody responses of vaccinated and unvaccinated goats. The first approach involved screening a phage expression library with pooled serum from infected goats, identifying previously reported *Map* antigens, including MAP_1272c and MAP_1569. However, three specific antigens detected only by vaccinated goats were also identified in the library screens. A second approach using dot blot analysis identified two additional differentially reacting proteins in the vaccinated goats (MAP_4106 and MAP_4141). These immunological results, combined with the microbiological and pathological findings obtained previously, provide a more complete picture of Johne’s disease control in goats vaccinated against *Map*.

## 1. Introduction

Johne’s disease is a chronic intestinal inflammation in ruminants that leads to malabsorption of nutrients. Because vaccination against Johne’s disease is seen as the most logical intervention method due to several inherent challenges, the Johne’s Disease Integrated Program (JDIP) conducted a multi-institutional vaccine study to evaluate live attenuated *Mycobacterium avium* subsp. *paratuberculosis* (*Map*) mutants as vaccine candidates against this disease [1]. JDIP represented a coordinated agricultural project funded by USDA’s National Institute of Food and Agriculture and the JDIP vaccine trial began in August of 2008 with the idea of selecting the most promising live-attenuated vaccine strains of *Map*, the pathogen that causes Johne’s disease, and testing them in a standardized, coordinated fashion. Prior to this, investigators would simply test their own attenuated mutant (also known as vaccine strain) independently and had no other vaccine strains to benchmark their results. To circumvent this issue, investigators from around the world were asked to submit their best candidate mutant strains to a lead institution where blinding of the strains occurred prior to distribution to testing labs. A total of 22 mutants were submitted from 14 institutions and the study design comprised three distinct phases to identify which are the most efficacious vaccine strains.

The first phase measured *Map* attenuation in bovine macrophages [2], the second phase measured *Map* colonization in mice [3] and the third phase measured protection from *Map* challenge in goats [4]. Each of these phases were all performed by one or two independent laboratories, which provided the needed standardization. Live attenuated mutant strains were triaged based on the results of the macrophage and mouse studies. To conclude this multi-year study, a final vaccine trial was conducted by testing the remaining five vaccine candidates in a goat challenge model [5] with protection metrics determined by skin testing, fecal shedding, lesion scores and tissue colonization [4].

A total of eighty 2-month-old goat kids were enrolled in the year-long study [4]. The study design included ten goats per vaccine strain with three additional 10-goat groups for controls. The commercial Johne’s vaccine, Silirum from Zoetis, was among the treatment groups. A type II bovine strain of *Map* was used for challenge as goats appear more susceptible to *Map* infection regardless of the strain used [6]. Total *Map* burden in the Hines et al. [4] study goats was measured by shedding (fecal PCR and fecal cultures) as well as tissue histopathology and culture. A similar study used the same goat model to evaluate live *Map* mutant strains and found reduced colonization along with a strong interferon gamma (IFN-γ) response in the vaccinated goats [7]. They further identified gene expression differences in peripheral blood mononuclear cells (PBMCs) from infected and vaccinated goats, although Mycopar was used as the commercial vaccine [8]. Other goat vaccine studies have observed immunological anergy to *Map* as disease progresses [9].

Additional details of the Hines et al. goat trial were described previously [4], but a key finding from that study was a strong protective response observed in the commercial vaccinated goats compared to the *Map* attenuated vaccine strains. Specifically, the commercial vaccine group showed the least number of lesions and no obvious thickening of the small intestine. There were also lower numbers of acid-fast bacilli in intestinal tissues and associated lymph nodes. Finally, shedding of *Map* in the feces was lowest in the commercial vaccine group [4]. Unfortunately, cell-mediated immune parameters or cytokine levels were not reported in the Hines et al. study [4]; however, it was of keen interest to capture any correlates of protection afforded by administration of the commercial vaccine. Therefore, in this study we closed this knowledge gap by examining vaccine-induced immunological parameters that resulted in low *Map* colonization in commercially vaccinated goats enrolled in the Hines et al. study [4] by measuring IFN-γ production, T cell population levels, and antibody profiles through expression library screening and dot blot analysis.

It is well-established that a strong, protective cell-mediated immune response along with proinflammatory cytokine secretion prevents full blown clinical Johne’s disease in cattle [10,11]. Leveraging important immunological data to this comprehensive vaccine study will yield a better understanding of the immune-based protection obtained with the commercial vaccine. We focused our study on three treatment groups from the Hines et al. study [4], which included the noninfected control group, the infected-no vaccine group, and the Silirum vaccinated group. Only the final 13-month post-challenge time point of the JDIP goat vaccine trial was examined. Notable immunological differences were identified in the vaccinated goats that might be attributed to the elevated protection against the disease as reported by Hines et al. [4].

## 2. Materials and Methods

### 2.1. Animals and Treatment Groups

A vaccine trial consisting of eight groups of ten kid goats per group was conducted in a previous study [4]. Whole blood and serum samples from three of those eight groups were analyzed in the present study. They include goats that were not vaccinated or infected (fed pasteurized goat milk only), infected only group (orally with goat milk containing two-consecutive daily 100 mg pelleted wet weight of *Map* (~10^9^ CFU) at age of 11 weeks), and the third group vaccinated with Silirum (Zoetis) once at 8 weeks of age and challenged with *Map* as above 3 weeks later [4]. Hereafter, these groups are designated as control, infected, and vaccinated, respectively. Goats were euthanized and necropsied at 13 months post-challenge. Animal handling, ACUC protocols and sampling were all performed in the previous study [4]. No animal handling was conducted in the current study.

### 2.2. Interferon Gamma Assay

Blood samples from ten goats in each of the three treatment groups were obtained from Dr. Murray E. Hines at the Tifton Veterinary Diagnostic Laboratory in Georgia and immediately shipped to the USDA-National Animal Disease Center. Whole blood from these animals were collected at the 13-month post-challenge end point and processed immediately for IFN-γ stimulation. Briefly, 300 μL of heparinized whole blood (containing ~606,000 polymorphonuclear cells) was dispensed in 96-well round bottom plates and stimulated with *Map* sonicated extract (MPS), Pokeweed mitogen (PWM), and no stimulation control (NS). The final concentration of each stimulant was 3 μg/well. Antigen stimulations were performed for 18-h at 39 °C in 5% CO_2_. The plates were centrifuged to pellet red cells and harvested plasma was stored at −20 °C. Secreted IFN-γ was measured by ELISA using the Bovigam TB kit (ThermoFisher Scientific, Waltham, MA, USA). Each sample was measured in duplicate and averaged.

### 2.3. Cell Culture

PBMCs were isolated from the buffy coat fractions of blood. PBMCs were resuspended in complete media [RPMI-1640 (Gibco, Grand Island, NY, USA) with 10% fetal calf serum (Atlanta Biologics, Atlanta, GA, USA), 100 U of penicillin G sodium (Gibco) per mL, 100 μg of streptomycin sulfate (Gibco) per mL, 0.25 μg of amphotericin B (Gibco) per mL, and 2 mm l-glutamine (Gibco)]. Cells were cultured at 2.0 × 10^6^/mL in replicate 48-well flat-bottomed plates (Corning Incorporated, Corning, NY, USA) at 39 °C in 5% CO_2_ in a humidified atmosphere. Duplicate wells were set up for each animal for the following in vitro treatments: medium only (no stimulation, NS), concanavalin A (ConA; 10 µg/mL; Sigma), PWM (10 µg/mL; Sigma) and MPS (10 µg/mL). Plates were incubated for either 3 days (NS, ConA, and PWM) or 6 days (NS, MPS) and cells were harvested for flow cytometric analyses. Freshly isolated PBMCs were also analyzed on the date of purification.

### 2.4. Flow Cytometry Assay

Three- or six-day PBMC cultures in 48-well plates were centrifuged at 1500 rpm for 5 min and the supernatant removed. Cells in the well bottom were gently resuspended in 300 μL of phosphate-buffered saline (PBS; 0.15 M, pH 7.4). Then, 50 μL of the cell suspension was transferred to 96-well round bottom plates (Corning Incorporated, Corning, NY, USA) and 50 μL of appropriately diluted primary monoclonal antibody to CD1, CD4, CD8, γδ T cells, B cells, CD14, as well as memory/activation markers, CD25, CD28, CD44, CD62L, and CD45RO was added to wells (VMRD, Pullman, WA; Washington State Monoclonal Antibody Center, Pullman, WA, USA). Antibody specificities and concentrations used are listed in Table 1. All wells received 10 μg/mL of DAPI (4′-6-diamidino-2-phenylindole; Sigma) or zombie yellow to differentiate live from dead cells and allow gating on viable cells. Cells were incubated at 4 °C for 30 min. and then plates were centrifuged at 1250 rpm for 2 min at 4 °C and the supernatant decanted. In total, 100 μL of secondary antibody cocktail consisting of fluorescein-conjugated anti-mouse IgM (Southern Biotech, Birmingham, AL, USA), R-phycoerythrin-conjugated goat F(ab)_2_ anti-mouse IgG_2a_ (Southern Biotech, Birmingham, AL, USA), and peridinin-chlorophyll-protein complex-conjugated rat anti-mouse IgG_1_ (Becton Dickinson, San Jose, CA, USA) diluted 1:312, 1:625, and 1:42, respectively, in PBS with 1% fetal calf serum and 0.04% sodium azide was added to designated wells and the plate was centrifuged again at 1250 rpm for 2 min at 4 °C. The cells were then suspended in 200 μL of BD FacsLyse (BD Biosciences, San Jose, CA, USA) for immediate flow cytometric analysis. Samples were evaluated using 30,000 events per sample on a FACScan flow cytometer (Cell Quest Software; BD Biosciences). Analysis was restricted to gating on viable mononuclear cells based on forward and side scatter characteristics of surface marker expression (FlowJo, Tree Star, Inc., San Carlos, CA, USA). 

### 2.5. Map-Lambda ZAP Expression Library Construction and Screening

The *Map* ATCC19698 expression library was prepared and used as previously described [12]. This strain is a type II bovine strain similar to the challenge strain. *E. coli* XL1-Blue MRF’ was used as the host strain for infection with the lambda ZAP phage clones containing *Map* genomic DNA fragments, which was plated at a MOI of 10:1 on 150 mm Petri plates containing NZY agar (3% [wt…vol^−1^] N-Z Amine A [Sigma Chemical Co., St. Louis, MO, USA], 1% [wt…vol^−1^] yeast extract [Fisher Scientific], pH 7.5). After 18 h at 37 °C to achieve full plaque development, duplicate 150 mm nitrocellulose filters were applied to each plaque-loaded plate and incubated in a blocking buffer consisting of PBS with 2% bovine serum albumin (BSA) and 0.1% tween 20 (PBS-BSA-T). The first lift was applied to a pre-chilled plate for 5 min and the second lift was applied for 20 min. These duplicate “plaque lifts” created a mirror image of all the plaques on each plate. Orientation marks on each membrane were created with needle punctures that also pierced the agar plate. These punctures later helped to orient the plate for picking positive plaques. 

Pooled sera were first absorbed with *E. coli* extract prior to use in screening the phage library. These extracts were prepared by centrifugation of log-phase *E. coli* XL1-Blue MRF’ cells and resuspended in dH_2_O at 1/10th the culture volume. The concentrated cells are sonicated for 1 min at 50% power, followed by a low-speed centrifuge (4000× *g*; 5 min) to remove large debris. The absorption reaction was 1:1 serum-to-extract conducted at room temperature for 1 h with gentle agitation. The sera-extract mixture was centrifuged at 13,000× *g* for 20 min to remove immune complexes. The first nitrocellulose filter was exposed to pooled preabsorbed goat sera from the commercial vaccinated goats and the second, duplicate filter was exposed to the infected group. Sera from all animals within each group were mixed equally and then diluted 1:200 in PBS-BSA-T. Plaques reacting with pooled sera from the vaccinated group, but not the infected group were designated gv-1 through gv-11 (goat vaccinated clone 1…11). These positive plaques were purified by repeated plating until a well-separated plaque could be cored and placed in phage buffer (50 mM Tris-HCl, 8 mM MgSO_4_·7 H_2_O, 100 mM NaCl, and 0.01% gelatin, pH 7.5). Phage were then subcloned by co-infection of *E. coli* XLOLR with the lambda phage lysate obtained from the positive plaque and the addition of ExAssist helper phage. This infection resulted in the in vivo excision of pBK-CMV phagemid containing *Map* genomic inserts. The rescued *E. coli* subclones were plated and selected on NZY agar with kanamycin at 50 µg/mL.

### 2.6. SDS-PAGE Gel Electrophoresis and Electrotransfer of Proteins

IPTG-induced XLOLR cells containing pBK-CMV clones were boiled for 5 min in SDS-PAGE loading dye (Bio-Rad Laboratories, Richmond, CA, USA). Sodium dodecyl sulfate-polyacrylamide gel electrophoresis (SDS-PAGE) was performed using 12% (*w/v*) polyacrylamide gels. Electrophoretic transfer of proteins onto pure nitrocellulose was accomplished with the Bio-Rad Trans-blot Turbo transfer system (Bio-Rad) and nitrocellulose transfer packages (Bio-Rad). The 5 min transfers were performed using the turbo blot setting. After transfer, filters were blocked with PBS-BSA-T and stored at 4 °C until ready to use.

### 2.7. Dot Blot and Immunoblot Assays

The dot blot assay was conducted similar to that described previously [13], but with the following modifications. PBS was used as the spotting buffer and diluent for all *Map* recombinant proteins purified previously [14] as well as the *Map* sonicated extract. Nitrocellulose membranes, pre-soaked in PBS, were placed inside a Bio-dot 96-well manifold apparatus (Bio-Rad) with no air bubbles trapped between the gasket and membrane. Each well of the Bio-dot was pre-washed with 200 μL of PBS followed by a brief vacuum to drain the wells. Purified recombinant maltose binding protein (MBP) fusions representing *Map* proteins were stored in deep 96-well plates (Nalgene Nunc International, Penfield, NY, USA) at a stock concentration of 0.5 μg/mL. Protein spotting was performed using a 12-channel multi-pipette such that each well was loaded with 200 μL of the protein-PBS stock, giving a final concentration of 0.2 μg protein/spot. Another brief vacuum was applied until proteins were impinged on the membrane. Then, each well was washed in 200 μL of PBS with 0.1% Tween-20 (PBS-T). The vacuum was re-engaged briefly until all fluid passed through the membrane and it was immediately removed from the apparatus and placed in a Petri dish containing PBS-BSA-T, a blocking solution. After 1 h in PBS-BSA-T, the immunoblotting assay was performed as described below. MBP-LacZ was included as a control to assess immunoreactivity to the affinity tag. A whole cell sonicated extract was also included as a control. Dot blot arrays were produced in sets of five with one of the arrays used as a spotting control, which was probed with the monoclonal antibody to MBP (α-MBP). This antibody was produced in our laboratory [15] and diluted 1:10,000 in PBS-BSA-T. The remaining four membranes were probed with two goat serum samples. 

For the immunoblot assays, sera from goats were preabsorbed with a high-density *E. coli* XLOLR cell extract, prepared as described above. Absorbed sera were then diluted 1:200 and used in Western blotting against whole cell antigen as well as recombinant *Map* proteins purified previously [14]. However, sera were not pre-absorbed for the dot blot assays. Sera were diluted in PBS-BSA-T and exposed to the blot at room temperature for 2 h. After three washes in PBS-T, blots were incubated for 1.5 h in peroxidase anti-goat IgG (H + L) from Vector Laboratories diluted 1:20,000 in PBS-BSA-T. Nitrocellulose blots were again washed three times (5 min each) in PBS-T as before and developed using SuperSignal PicoWest Plus chemiluminesent substrate detection reagents (Thermo Scientific).

Dot blot intensity measurements were conducted using the Adobe Photoshop CS6 extended version 13.0.6 measurement tool. The grayscale image was inverted and levels checked to ensure all pixels were between 0 and 255. Each spot along with its local background was measured and subtracted. Spots on the corresponding MBP-probed dot blot were measured in the same way and used to correct for relative amount of recombinant protein spotted using a method described previously [16].

### 2.8. Statistical Analysis

Cell population percentages obtained by flow cytometric analysis were analyzed by using the PROC Mixed analysis of SAS (PROC MIXED in SAS^®^ PC Windows Version 9.1.3 software). Values were reported as least square means ± standard errors of the mean unless noted otherwise. When significant effects (*p* < 0.05) due to vaccination, infection or in vitro treatment were noted, means comparison was conducted using the Tukey–Kramer post hoc test.

## 3. Results

### 3.1. Secretion of IFN-γ in Vaccinated, Infected and Control Goats

**Figure 1:**
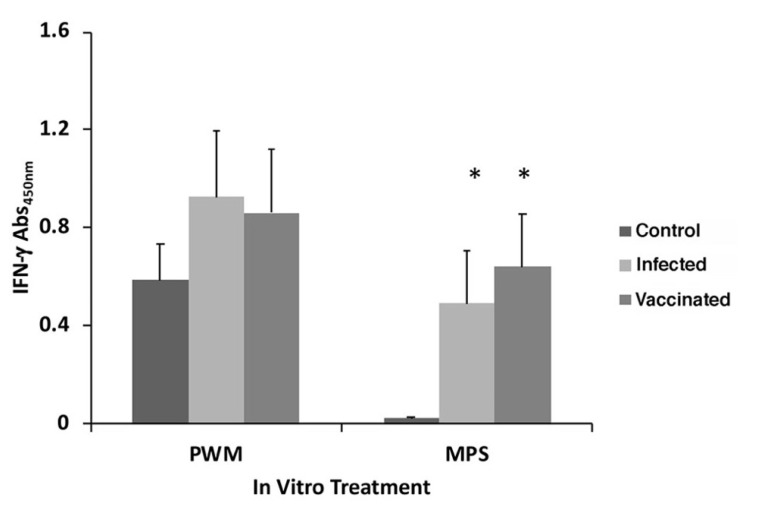
Secretion of IFN-γ from whole blood stimulated for 18-h in vitro with pokeweed mitogen (PWM) and *Map* sonicated extract (MPS). Data are the average of duplicate trials ± standard error of the mean. The treatment groups are shown at the right. The asterisk indicates a *p* value less than 0.05 compared to the control group.

Stimulation with PWM resulted in strong IFN-γ responses, however, they trended higher for the two groups, infected and vaccinated, versus the control group (Figure 1). Upon stimulation with MPS, negligible secretion of IFN-γ occurred in the control goats, as expected. However, equally strong antigen-specific IFN-γ secretion was observed for both treatment groups infected with *Map*, regardless of vaccination (Figure 1). 

### 3.2. T Cell Subpopulations

**Figure 2:**
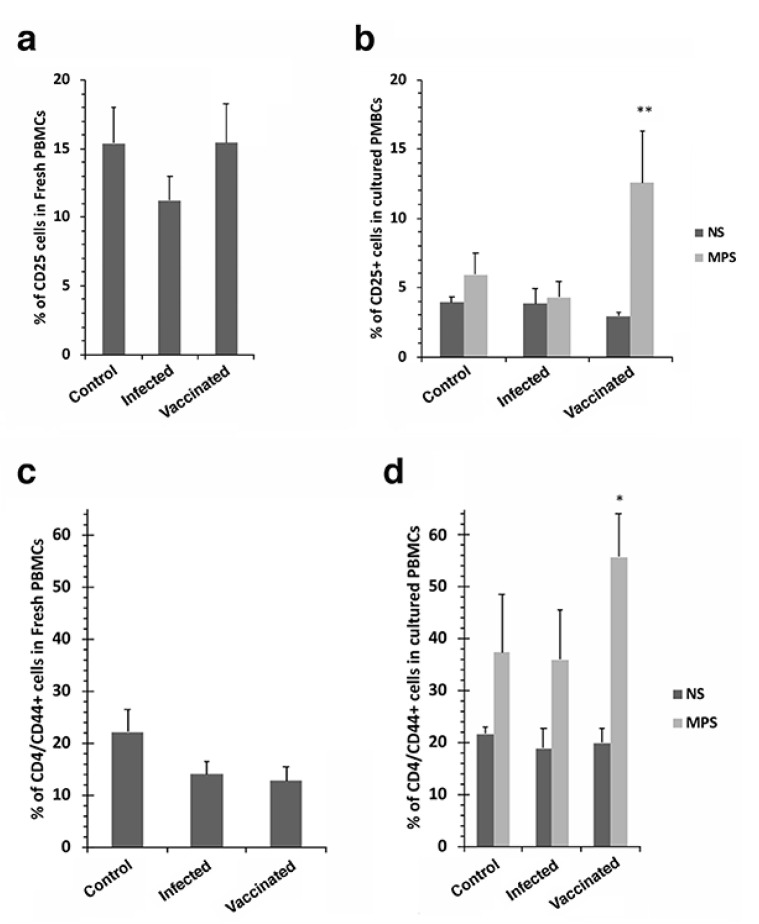
CD25+ and CD4/CD44+ T cell proliferation of cultured PBMCs in response to *Map* antigen. The percentage of CD25+ T cells in freshly isolated PBMCs are shown in (**a**) while cultured PBMCs from the same treatment groups are shown in (**b**). The same arrangement is shown for the CD44+ subpopulation of CD4 cells (**c**,**d**). Note the proliferation of CD25+ and CD44+ cells after stimulation with *Map* antigen (**b**,**d**). The double asterisk indicates a *p* value < 0.01 while the single asterisk indicates a *p* value < 0.05. Data are the average of duplicate trials ± standard error of the mean. The treatment groups are shown at the right of (**b**,**d**) (NS = no stimulation; MPS = stimulation with *Map* sonicate extract).

**Figure 3:**
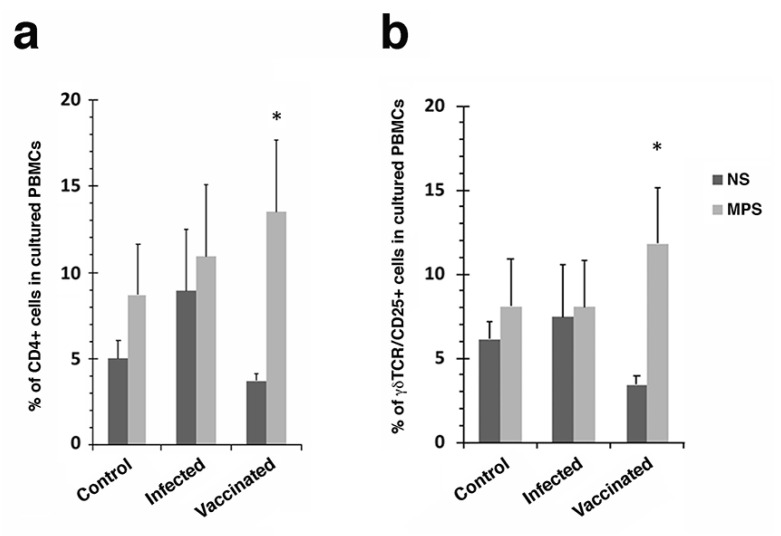
CD4+ and γδ-T cell proliferation of cultured PBMCs in response to *Map* antigen. The percentage of CD4+ (**a**) and γδ TCR/CD25+ cells (**b**) in cultured PBMCs are shown. Note the proliferation of each cell population after stimulation with MPS. Asterisk indicates a *p* value < 0.05. NS = no stimulation; MPS = stimulation with *Map* sonicate extract.

Significant increases in CD25+ and CD44+ T cells were observed in cultured PBMCs stimulated with *Map* antigen for vaccinated goats (Figure 2b,d). This same effect was not observed with fresh PBMCs (Figure 2a,c) or in the other treatment groups not receiving the vaccine. Other cell populations from vaccinated goats that showed a significant increase in response to *Map* antigen stimulation included CD4+ and γδ cells (Figure 3).

**Figure 4:**
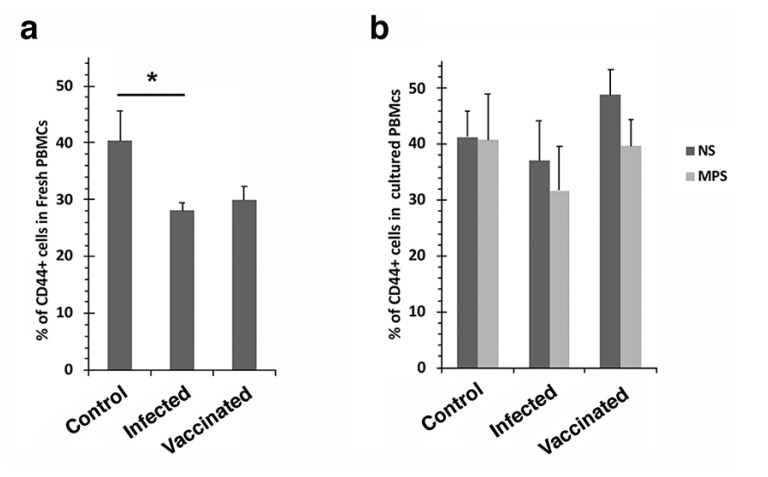
CD44+ T cell proliferation of fresh (**a**) and cultured PBMCs (**b**) in response to *Map* antigen. The percentage of fresh CD44+ decreased in the infected and vaccinated groups (**a**). No significant responses were observed in cultured PBMCs (**b**). Asterisk indicates a *p* value of less than 0.05. Treatments: NS = no stimulation and MPS = stimulation with *Map* antigen.

The CD4/CD62L+ populations decreased when stimulated with *Map* antigen for all treatment groups, although this effect was not significant (Supplemental Figure S1). CD44 and CD62L represent 2 types of memory effector cells and exposure to *Map* antigen in culture resulted in different patterns for the CD4+ T cells in the vaccinated group (Figure 2d and Supplemental Figure S1b). This divergent response may be explained by a switch from central memory to effector memory cells. No other significant differences were noted among the remaining T cell populations examined, including CD1+, CD14+ and CD45RO+ cells (Supplemental Figure S2). Furthermore, no significant differences were observed in any T cell populations when sorting fresh PBMCs, with the exception of CD44+ cells, which decreased significantly between control and infected groups (Figure 4a); furthermore, there was no significant response when stimulating with *Map* antigen (Figure 4b).

### 3.3. Map Antigens Detected Post Vaccination

**Figure 5:**
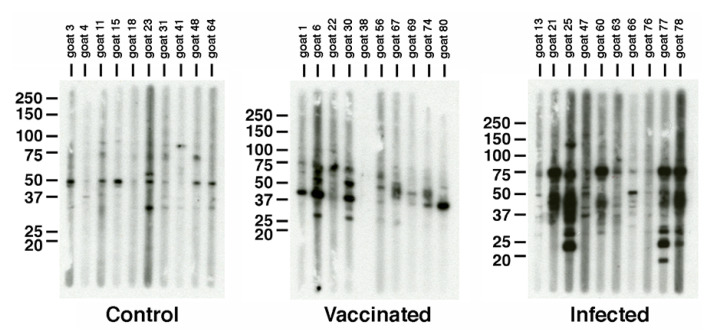
SDS-PAGE and strip immunoblot analysis of *Map* whole cell lysates. Preparative SDS-PAGE gels containing *Map* sonicated extract were immunoblotted. The blot was placed in a slot blot device and loaded with sera from each goat assigned to the three treatment groups. Note the variable antibody responses of each goat within the same treatment. Kilodalton size standards are indicated in the left margin and treatment is indicated beneath each immunoblot. All ten animals within each treatment were analyzed.

We next characterized the goat antibody response to the vaccine. It was of interest to identify if any *Map* antigens are detected in goats vaccinated with the commercial vaccine compared to the infected and control groups. Individual sera from each goat in the 3 treatment groups were analyzed by strip Western blots. In general, antibodies labeled a variety of *Map* proteins regardless of treatment group; however, the control no challenge group showed very few reactive proteins (Figure 5, left panel). The strongest antibody response was noted for the infected group, which labeled proteins ranging from 15- to 100-kDa in size (Figure 5, right panel). Interestingly, the commercial vaccine showed very little antibody reactivity compared to the infected group, perhaps indicative of the lower *Map* burden demonstrated in the Hines et al. study [4]. Likewise, the breadth of protein sizes detected was comparatively narrow, ranging from 25- to 75-kDa. These results suggested that different antigens might be prominent in the vaccinated group compared to the infected group.

#### 3.3.1. Map-Phage Expression Library Screen to Identify Reactive Antigens

**Figure 6:**
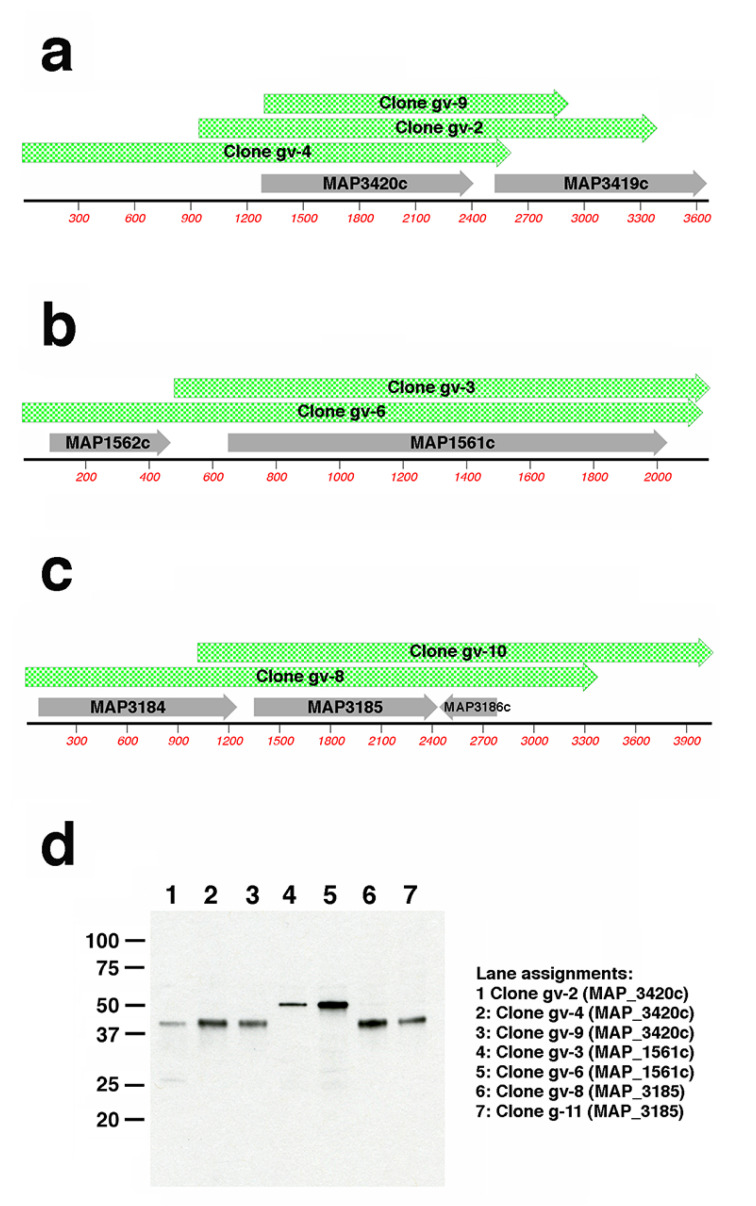
MAP_1561c, MAP_3185, and MAP_3420c are detected in vaccinated goats. Sequence analysis of expression library subcloned inserts shows three (**a**), two (**b**), and two (**c**) overlapping sequences aligned with the corresponding K-10 genome sequence with annotated coding sequences shown in gray. Clone insert sizes and genome coordinates are shown in Supplemental Table S1. (**d**) Immunoblot analysis of IPTG-induced lysates harboring each clone in (**a**)–(**c**). Pooled goat sera diluted 1:400 served as the primary antibody. Kilodalton size standards are indicated in the left margin. Lane assignments are shown right of the panel (**d**) blot. Note that only MAP_3420c is common to all inserts in (**a**), only MAP_1561c is common to both inserts in (**b**) and only MAP_3185 is common and of the correct size for aligned inserts in (**c**).

To identify *Map* antigens detected in each group, a *Map*-lambda phage expression library was differentially screened with antisera from the two treatment groups. Duplicate nitrocellulose plaque lifts were produced to screen approximately 80,000 library clones. One nitrocellulose lift from each plate was probed with pooled sera from vaccinated goats and the other lift was exposed to pooled sera from infected goats. Over 100 positive plaques were detected by the infected goat sera, and among these, 39 plaques were detected by sera from both groups. Nine of these plaques reacting with sera from both infected and vaccinated goats were randomly selected for purification, subcloning and sequencing. Only 11 plaques were detected solely by sera from vaccinated goats and these were also plaque purified and sequenced. Analysis of these 20 positive phage clones showed an average insert size of 2.7 kb with the same genomic regions present in multiple clones (Supplemental Table S1). SDS-PAGE and immunoblot analysis of *E. coli* cultures harboring these 20 mycobacterial subclones showed that each produced a protein that was recognized by pooled sera from the vaccinated goats (Figure 6 and Supplemental Figure S3) or by goats from both groups (Supplemental Figures S4 and S5). The combined sequence and immunoblot analysis revealed the identity of novel antigens, detected only by the vaccinated goats, encoded by MAP_0585, MAP_1561c, MAP_3185 and MAP_3420c (Figure 6 and Supplemental Figure S3). 

Conversely, well-known *Map* antigens were detected by sera from both infected and vaccinated goats. These include MAP_1569 (modD), which was identified in 7 of the 9 clones (Supplemental Figure S4). This gene encodes ModD, which has been identified as a strong antigen in cattle with Johne’s disease [17,18]. The other two clones contained MAP_1272c (Supplemental Figure S5), which has also been previously identified as a strong *Map* antigen [19]. A summary of the antigens detected in each library clone is shown in Table 2. 

#### 3.3.2. Dot Blot Analysis to Identify Reactive Antigens

**Figure 7:**
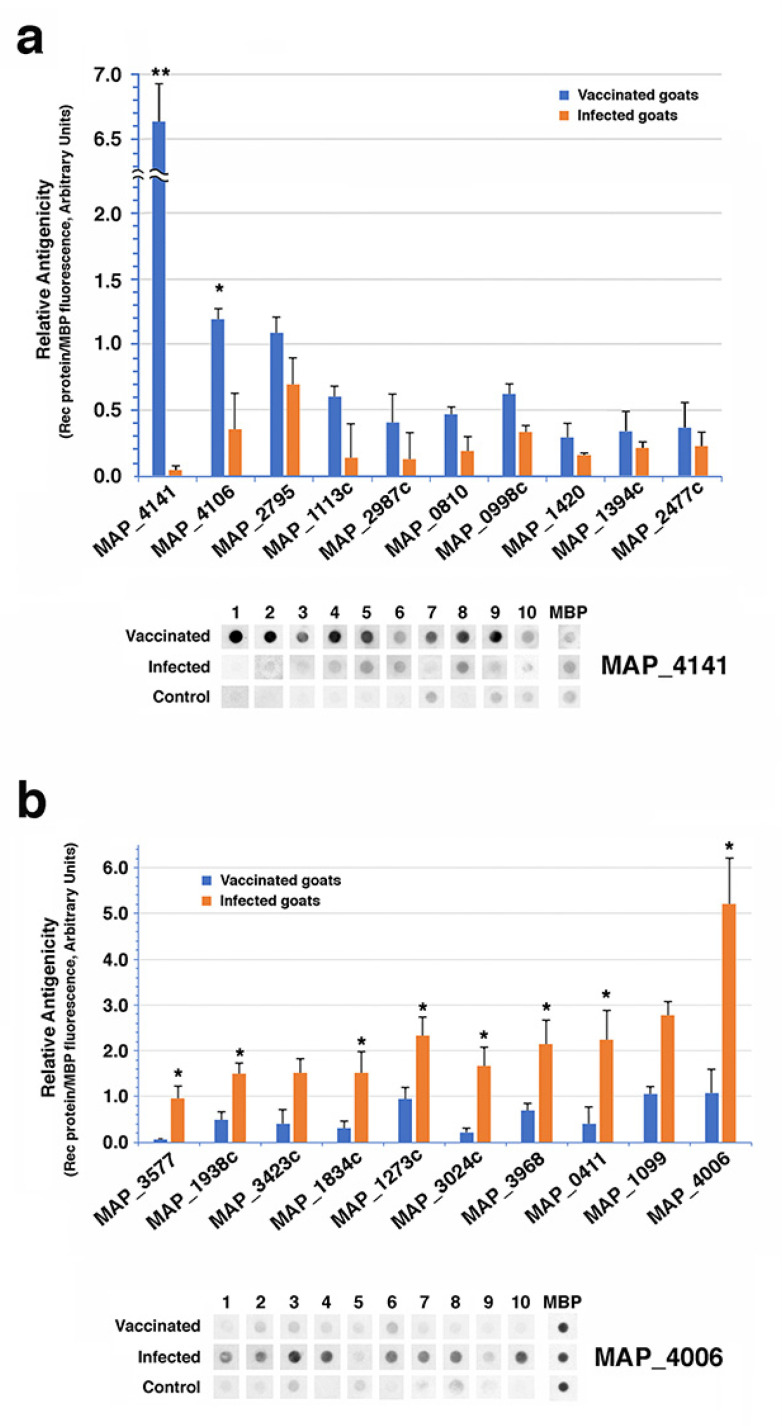
Antibody profiling of infected and vaccinated goats using 190 recombinant proteins. (**a**) The ten most differentially reactive recombinant proteins in the vaccinated group. Note the *y*-axis scale break for MAP_4141. (**b**) Ten most differentially reactive proteins in the infected group. Error bars represent standard error of the means. Asterisks denote significance between treatments (* = *p* < 0.05; ** = *p* < 0.01). Beneath each bar graph is the dot blots of all 30 goats for the strongest antigen in that treatment group. The MBP spots indicate amount of recombinant protein spotted.

As an alternate method to profile antibody differences between infected and vaccinated goats, dot blots of 190 recombinant proteins were probed with sera from two goats in each treatment group (Supplemental Table S2). These results showed additional differ- ences in the antibody response to *Map* in a subset of goats from each treatment group. A total of 37 recombinant proteins were differentially recognized by sera from vaccinated goats, but only two were significant. The strongest differential antibody levels between the vaccinated goats and the infected goats were observed with a 30S ribosomal protein S7 encoded by MAP_4141 (Figure 7a) while MAP_4106 was the only other protein showing significantly higher antibody levels in vaccinated goats. Conversely, eight antigens showed significant differential antibody levels in the infected goats (Figure 7b).

The only *Map* protein present on the dot blots that was also identified from the phage library screen was MAP_3185, a PPE family protein. This protein was detected by the vaccine only goats in the library screen (Table 2) and dot blot analysis confirms stronger reactivity to the vaccinated goats compared to infected goats (Supplemental Table S2).

## 4. Discussion

In the study by Hines et al., it was observed that the commercial vaccine outperformed all five of the live-attenuated vaccines based on fecal shedding, microscopic examination of tissues and culture data [4]. A goal of this study was to determine if there are any immunological parameters that might correspond to the decreased disease signs observed in the commercially vaccinated goats. From previous studies, a strong protective Th1 cell-mediated immune response best controls Johne’s disease but as disease progresses, a Th2 immune response becomes more predominant [20,21], which could be associated with immune tolerance to the pathogen [9]. An understanding of the triggers for this Th1/Th2 switch will provide critical insights for Johne’s disease control [22], but these triggers have yet to be identified. During clinical disease, Th1 immune cells that are present do not react upon known stimulants, suggesting a state of anergy for both cattle [23] and sheep [24]. Against this background we sought to determine if the vaccinated goats from the Hines et al. study [4] still possessed Th1 type immune cells since the disease was less severe in those animals. CD4+ T cells are the primary lineage of Th1 cells that secrete IFN-γ among other cytokines and they are prominent in ileum lesions [25]. Both infected and vaccinated goats from this study showed a significant increase in IFN-γ production compared to control goats (Figure 1). This shows a natural host response to the pathogen despite vaccination since lack of IFN-γ production correlates with disease development [26,27]. 

T cell subpopulations were also examined and showed that CD25+, CD44+ and γδ cells all significantly proliferated when vaccinated goats were stimulated with *Map* antigen (Figure 2 and Figure 3). However, there were no significant differences with CD1+, CD62L+, CD28+, and dendritic cells (Supplemental Figure S2). These results suggest that CD25+, CD44+ and γδ cells may play a more prominent role in defending the host from Johne’s disease progression than other cell types. These cell types are characteristic of responses to mycobacterial vaccines, providing immunoregulatory (CD25+) activity, memory (CD44+) responses to antigen, as well as a bridge between innate and adaptive immunity (γδ cells) [28,29,30]. Our previous study of the immune response to goats experimentally infected with mucosal scrapings of *Map* from bovine intestinal tissues [31] shows similar results to that presented herein. There was a steady decrease in IFN-γ production as disease progressed in the final 6 months of that year-long study, with similar IFN-γ levels observed at the 360-day time point [31]. Likewise, CD4+, CD8+ and γδ cells were all present in similar levels. However, CD25+ cells were not measured in that study. 

The commercial vaccine is composed of an inactivated strain designated 316F as indicated by the manufacturer, however, analysis by immunoblot against a whole cell extract of *Map* revealed very little antibody response at the time of necropsy compared to the infected goats (Figure 5). The general antibody response from vaccinated goats was characterized during the entire 13-month study by Hines et al. using a commercial ELISA test [4]. These data showed an antibody response that peaked at 3 to 5 months in goats vaccinated with the commercial vaccine compared to live attenuated vaccines, which peaked at or near the end of the study. In addition, none of the goats in the commercial vaccine group tested positive using the agar gel immunodiffusion assay. This result is consistent with goats vaccinated with another commercial vaccine, Mycopar, where the antibody levels were strong early, but continued to decline until the end of the year-long study [7]. We conclude that declining antibody responses in vaccinated animals do not reduce protection of the vaccine. Screening of the lambda phage library with pooled goat sera resulted in over 100 positive plaques, but most of those were detected by the infected goats. Only 11 positive plaques were specific to the vaccinated goats and from these, only 4 proteins were identified. These proteins include two PPE family proteins (MAP_3185 and MAP_3420c), a FAD-reductase (MAP_1561c) and a hypothetical protein (MAP_0585). 

A few known antigens were strongly overrepresented among the phage library clones reacting to both groups, including MAP_1272c and MAP_1569. Interestingly, both of these antigens appear to play an undefined role in invasion of bovine cells [32,33,34]. MAP_1569 is an alanine and proline rich antigen that has several aliases in the literature including ModD [35], FAP [34] and Apa protein [17,36]. Originally identified as a fibronectin attachment protein [33] and then a secreted antigen [35], this *Map* protein is routinely identified in antigen discovery approaches [17,18,37,38]. It is present in a molybdenum transport system operon and is upregulated in response to nutrient deprivation in *M. tuberculosis* [39]. MAP_1272c has also been extensively studied as an antigen [19,37,40] and functionally as a peptidoglycan hydrolyase [41]. This antigen was recently incorporated into a promising subunit vaccine for Johne’s disease [42].

Dot blot analysis of over 90 *Map* recombinant proteins with sera from vaccinated and infected goats also showed a more robust antibody response to defined *Map* proteins in the infected group. The antibody profiles between treatment groups were similar overall with only 37 recombinant proteins shown to be differentially recognized by sera from vaccinated goats and of these only two were significant. Conversely, 153 proteins were more strongly reactive in the infected goats, with eight significantly different.

Only MAP_3185 was detected by vaccinated but not infected goats in both the library screen and dot blot approaches. This antigen is a PPE family protein containing the SVP motif. The function of these proteins is unknown, but it has been proposed that they contribute to antigenic variation or virulence [43]. The general lack of correlation is explained by the fact that only 190 recombinant proteins were assayed by dot blot, while thousands of proteins were screened in the phage expression library. However, MAP_4141 theoretically should have been identified in the differential library screen. In checking the coding sequence of MAP_4141, there are three Sau3AI restriction sites, which is the enzyme used to partially digest the genomic DNA for preparing the library. While this might seem more than expected in a gene less than 500 bp, it is within the range of other genes identified from this library previously [44]. The other possibility is that although MAP_4141 is clearly differentially antigenic among this set of 190 recombinant proteins, if analyzed against a complete set of 3500 proteins produced by *Map*, it might not be among the top antigens. It remains unclear why a ribosomal subunit protein would elicit more antibody in vaccinated goats than in infected goats. 

In summary, the overall immune profile is very similar between the vaccinated and infected goats; however, defined changes in the T cell subpopulations and antibody response have been identified in the vaccinated goats. These studies yield solid hints at candidates for a subunit vaccine as well as an understanding of cell types necessary for disease reduction. Furthermore, the immunological analysis of goats vaccinated in the JDIP study has provided a clearer picture of the goat immune response correlates that lower *Map* shedding and presence in tissues. 

## 5. Conclusions

This study suggests that a protective response in goats may originate in the proliferation of certain immune cell types, including CD25+, CD44+ and γδ cells. The divergent responses of memory cells may suggest a regulation of inflammation. Although a different antibody profile was determined for vaccinated goats, its role, if any, in protection from disease remains unclear. It is likely that additional immune factors, beyond those uncovered in this study, play a beneficial role in protection against Johne’s disease. 

## Figures and Tables

**Table 1:** Primary antibodies used in flow cytometry experiments ^a^.

Antigen	mAb Clone	Isotype	Working Concentration ^b^ (μg/mL)	Specificity
CD1	TH97A	IgG2a	10	Dendritic cell
CD4	GC50A	IgM	14	T-helper cell
CD8	CACT80C	IgG_1_	14	T-cytotoxic/suppessor cell
N12	CACTB6A	IgM	14	γδ-cell receptor
B cell	GB26A	IgM	7	Total B cell
CD14	CAM66A	IgM	10	Monocyte
CD25	CACT116A	IgG_1_	15	Regulatory marker
CD28	TE1A	IgM	15	Activation marker
CD45RO	GC42A	IgG_1_	10	Memory/activation marker
CD44	BAG40A	IgG_1_	10	Memory marker
CD62L	DUI-29	IgG_1_	10	Memory marker
CD172a	DH59B	IgG_1_	10	Dendritic cells

^a^ Washington State University Monoclonal Center (Pullman, WA). ^b^ Diluted in PBS with 1% fetal calf serum and 0.04% sodium azide.

**Table 2:** Antigens identified from 20 expression library subclones.

Clone	Treatment	Antigen	Description	Size (kDa)
g1	Both	MAP_1569 ModD	Hypothetical protein	36.1
g2	Both	MAP_1569 ModD	Hypothetical protein	36.1
g3	Both	MAP_1569 ModD	Hypothetical protein	36.1
g4	Both	MAP_1272c	Hypothetical protein	29.2
g5	Both	MAP_1569 ModD	Hypothetical protein	36.1
g6	Both	MAP_1569 ModD	Hypothetical protein	36.1
g7	Both	MAP_1569 ModD	Hypothetical protein	36.1
g8	Both	MAP_1569 ModD	Hypothetical protein	36.1
g9	Both	MAP_1272c	Hypothetical protein	29.2
gv-1	Vaccinated	MAP_0585	Hypothetical protein	34.4
gv-2	Vaccinated	MAP_3420c	PPE family protein	38.6
gv-3	Vaccinated	MAP_1561c	FAD-dependent oxidoreductase	49.6
gv-4	Vaccinated	MAP_3420c	PPE family protein	38.6
gv-5	Vaccinated	MAP_0585	Hypothetical protein	34.4
gv-6	Vaccinated	MAP_1561c	FAD-dependent oxidoreductase	49.6
gv-7	Vaccinated	MAP_0585	Hypothetical protein	34.4
gv-8	Vaccinated	MAP_3185	PPE family protein	36.4
gv-9	Vaccinated	MAP_3420c	PPE family protein	38.6
gv-10	Vaccinated	MAP_3185	PPE family protein	36.4
gv-11	Vaccinated	MAP_0585	Hypothetical protein	34.4

## Data Availability

All data are included in the article and Supplementary Materials. Further inquiries can be directed to the corresponding author.

## Supplementary Materials

The following are available online at https://www.mdpi.com/article/10.3390/vaccines10040518/s1, Figure S1: The percent of CD62L+ cells in cultured PBMCs, Figure S2: The percent of CD1+ and CD45RO+ cells in cultured PBMCs, Figure S3: MAP_0585, a gene encoding a hypothetical protein, is detected in vaccinated goats, Figure S4: MAP_1569 is detected in vaccinated and infected goats, Figure S5: MAP1272c is detected in vaccinated and infected goats, Table S1: Sequence data of subcloned inserts obtained from the *Map*-lambda phage expression library, Table S2: Relative antigenicity of recombinant proteins probed with two infected goats and two vaccinated goats.
