# Supplementary material for: Immunological Evaluation of Goats Immunized with a Commercial Vaccine against Johne’s Disease
Source: Vaccines (Basel). 2022 Mar 26;10(4):518. doi: 10.3390/vaccines10040518 (PMC9031733; doi:10.3390/vaccines10040518)
Supplement: Supplementary file 1 [file vaccines-10-00518-s001.zip › vaccines-1605863-supplementary.pdf]

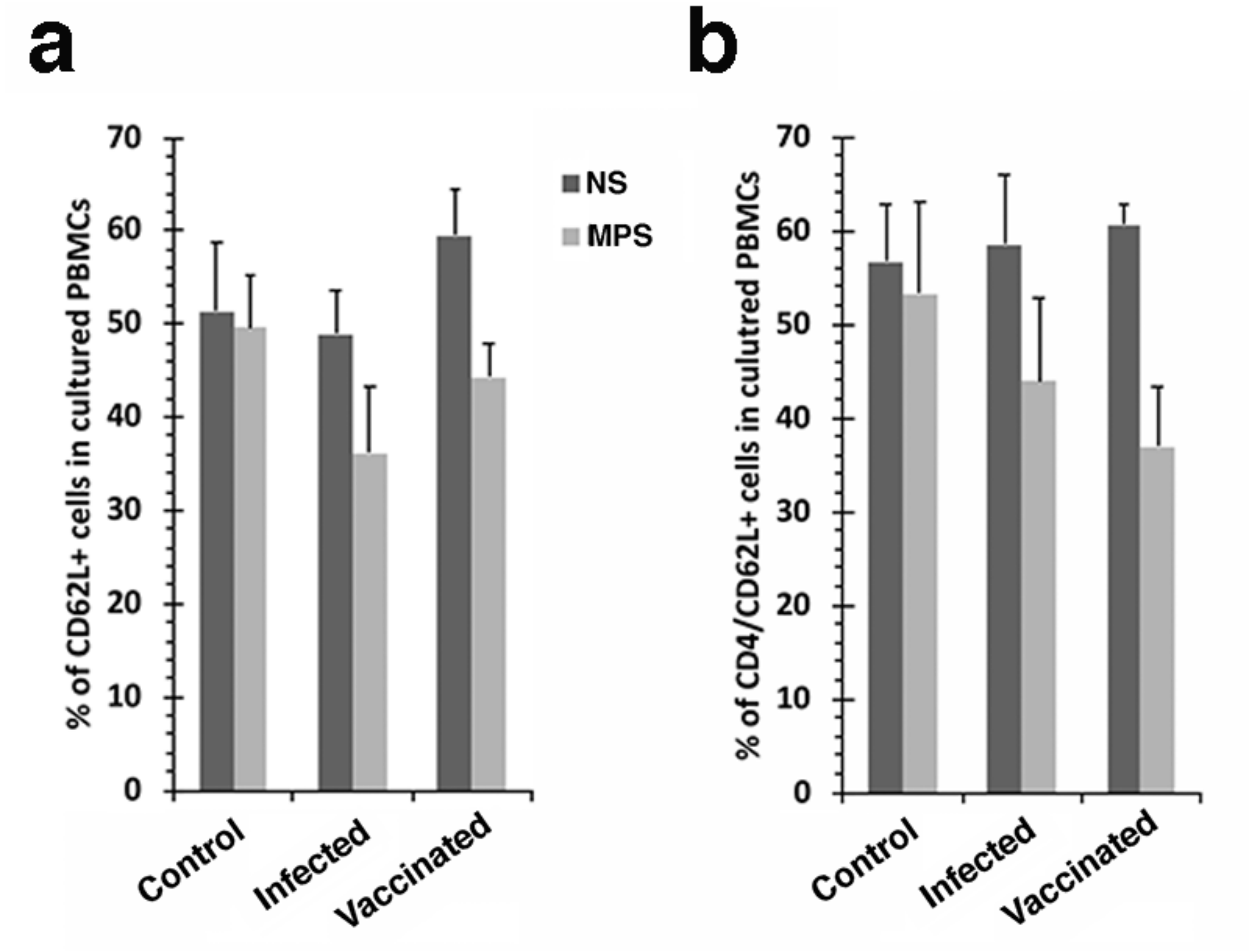

Supplemental figure S1: The percent of CD62L+ cells in cultured PBMCs are shown (**a**) along with the CD62L+ subpopulation of CD4+ cells (**b**). Stimulated with *Map* antigen consistently lowered the presence of these cell types, but the effect was not significant. The treatments are shown between **a** and **b** (NS=no stimulation and MPS=stimulation with *Map* antigen).

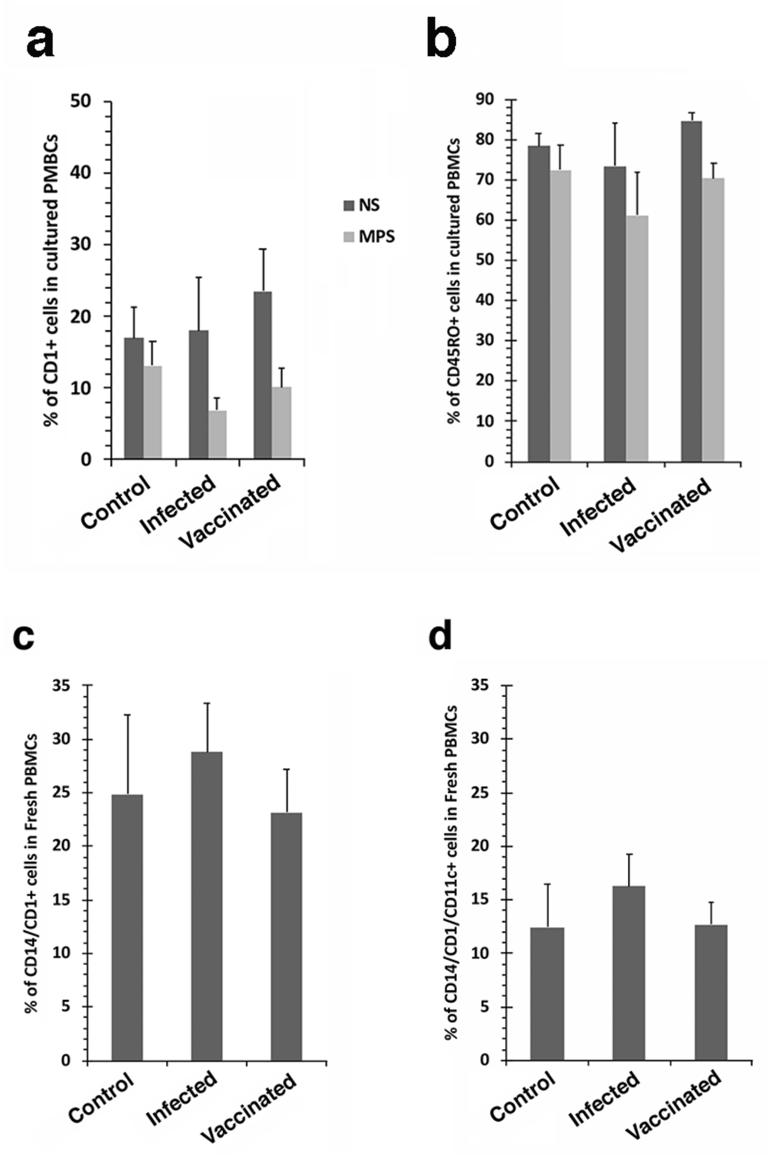

Supplemental figure S2: The percent of CD1+ (**a**) and CD45RO+ cells (**b**) in cultured PBMCs are shown. Stimulated with *Map* antigen consistently lowered the presence of these cell types, but the effect was not significant. The treatments are shown between **a** and **b** (NS=no stimulation and MPS=stimulation with *Map* antigen). Panels **c** and **d** examine dendritic cell populations in fresh PBMCs.

Supplemental Table S1. Sequence data of subcloned inserts obtained from the *Map*-lambda phage expression library

Clone Name	Treatment group	Annotated ORFs present in clone insert	K-10 Coordinates for: T3 end[1]	T7 end[1]	Insert size[2]
g1	Both	MAP1569 – MAP1571	1,723,219	1,725,800	2,582 bp
g2	Both	MAP1569 – MAP1571	1,722,980	1,725,785	2,806 bp
g3	Both	MAP1569 – MAP1571	1,723,219	1,725,939	2,721 bp
g4	Both	MAP1271c, MAP1272c	1,360,120	1,357,433	2,688 bp
g5	Both	MAP1569 – MAP1571	1,723,219	1,725,800	2,582 bp
g6	Both	MAP1568 – MAP1571	1,722,626	1,725,469	2,844 bp
g7	Both	MAP1569 – MAP1571	1,723,219	1,726,242	3,024 bp
g8	Both	MAP1568 – MAP1571	1,722,597	1,725,800	3,204 bp
g9	Both	MAP1271c, MAP1272c	1,360,120	1,357,004	3,117 bp
gv-1	Vaccinated	MAP0583 – MAP0586c	609,091	611,221	2,131 bp
gv-2	Vaccinated	MAP3419c – MAP3420c	3,799,578	3,797,133	2,446 bp
gv-3	Vaccinated	MAP1561c	1,714,775	1,716,462	1,688 bp
gv-4	Vaccinated	MAP3420c	3,796,315	3,798,923	2,609 bp
gv-5	Vaccinated	MAP0582 – MAP0586	607,731	611,591	3,861 bp
gv-6	Vaccinated	MAP1561c, MAP1562c	1,714,798	1,716,940	2,143 bp
gv-7	Vaccinated	MAP0583 – MAP0585	608,935	611,039	2,105 bp
gv-8	Vaccinated	MAP3184 – MAP3186c	3,533,908	3,537,283	3,376 bp
gv-9	Vaccinated	MAP3419c, MAP3420c	3,797,317	3,798,940	1,624 bp
gv-10	Vaccinated	MAP3185 – MAP3187	3,535,425	3,538,465	3,041 bp
gv-11	Vaccinated	MAP0585 – MAP0587	609,840	613,206	3,367 bp

[1] The front (T3) and back (T7) represent ends of the cloned insert relative to the lacZ promoter.

[2] K-10 genome coordinates were obtained from the RefSeq genome (GCF_000007865.1).

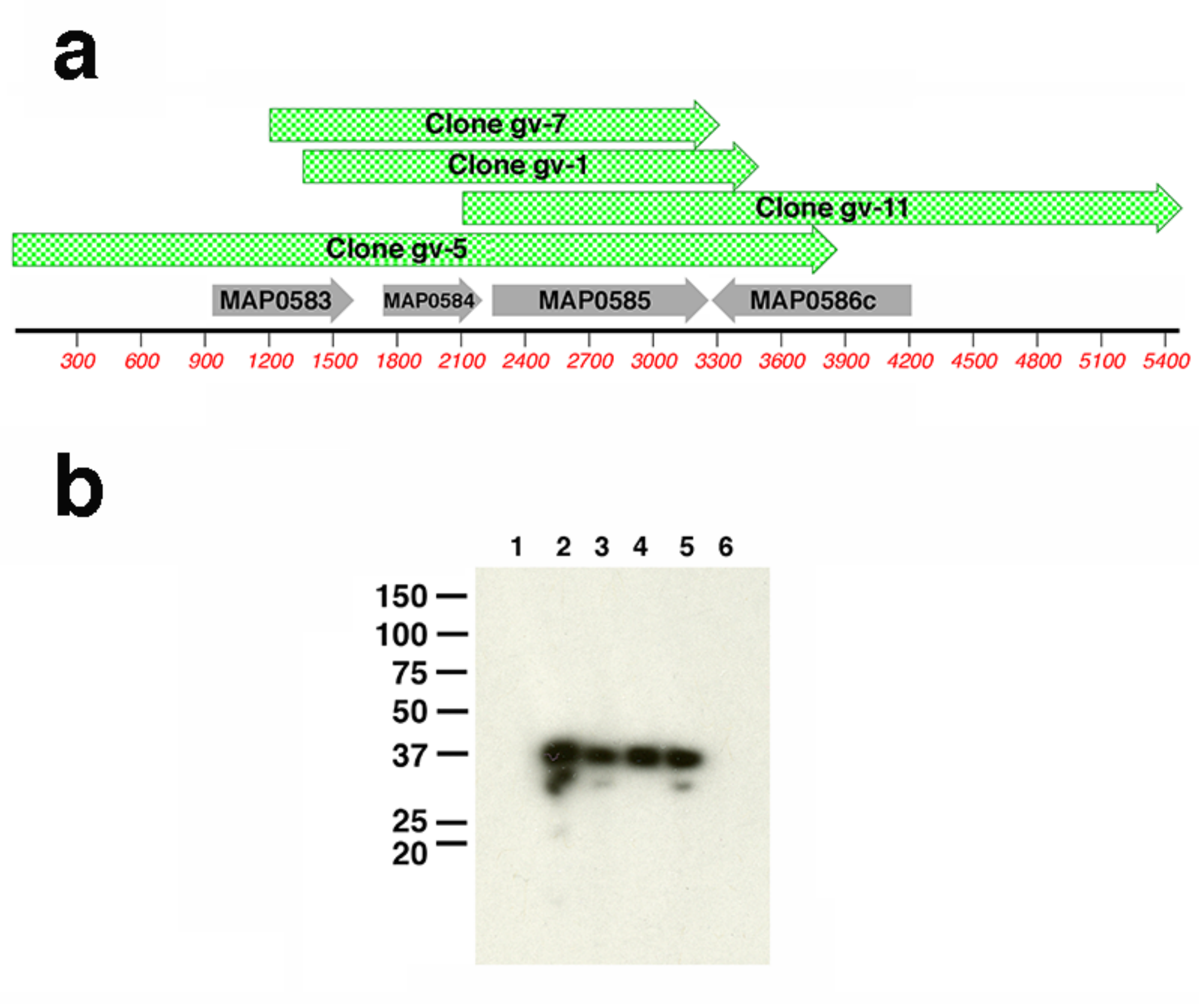

Supplemental Figure S3. MAP_0585, a gene encoding a hypothetical protein, is detected in vaccinated goats. Sequence analysis of expression library subclone inserts shows four overlapping sequences aligned with the corresponding K-10 genome sequence (**a**). Only the MAP_0585 gene was present in all four clones. Clone insert sizes and genome coordinates are shown in Table 1. (**b**) Immunoblot analysis of IPTG-induced lysates harboring each clone in (**a**). Pooled goat sera diluted 1:400 served as the primary antibody. A similar sized band approximately 35-kDa in size is detected in each sub-clone. Kilodalton size standards are indicated in the left margin. Lanes: 1=Protein size standards; 2=Clone gv1; 3=Clone gv5; 4=Clone gv7; 5=Clone gv11; 6=*E.coli* control lysate.

a

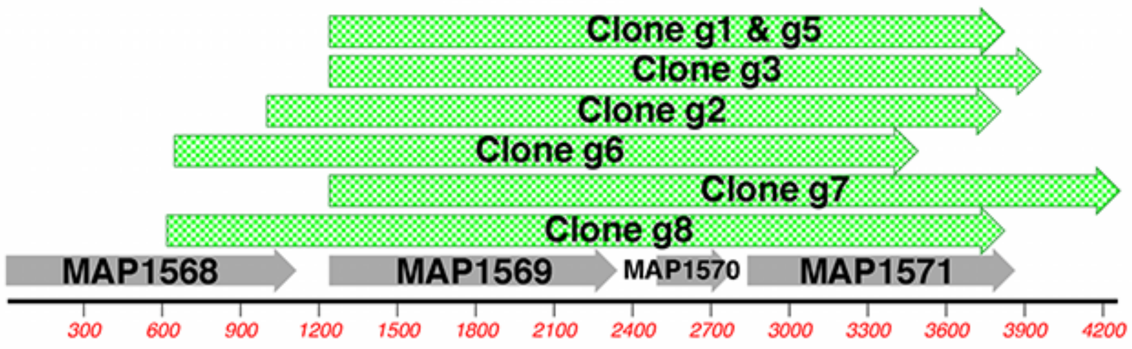

b

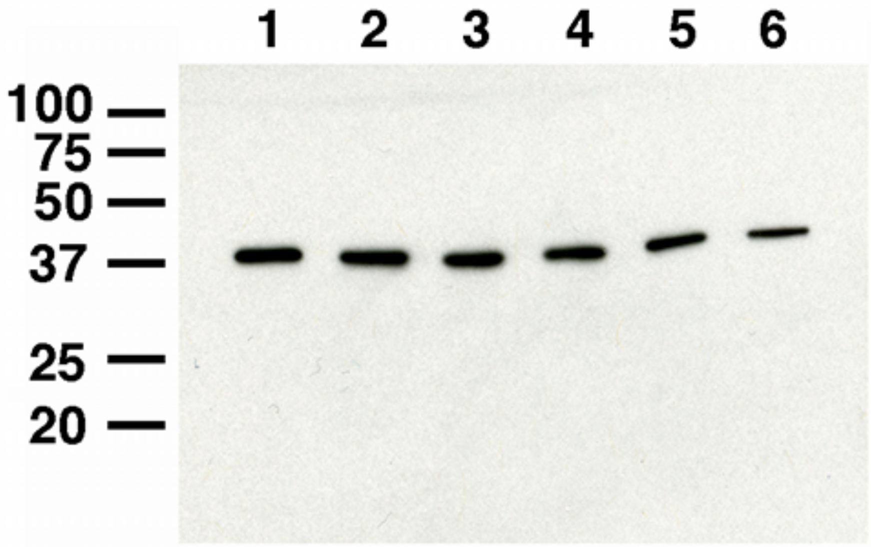

Supplemental Figure S4. MAP_1569 is detected in vaccinated and infected goats. (**a**) Overlapping subclone sequences aligned with the corresponding K-10 genome with annotated coding sequences shown in gray. (**b**) Immunoblot analysis of overlapping IPTG-induced lambda phage subclones shown in **a**. The blot was probed with pooled sera from the vaccinated goats. All cloned inserts contained the full length MAP_1569 and MAP_1570 genes. Kilodalton size standards are indicated in the left margin.

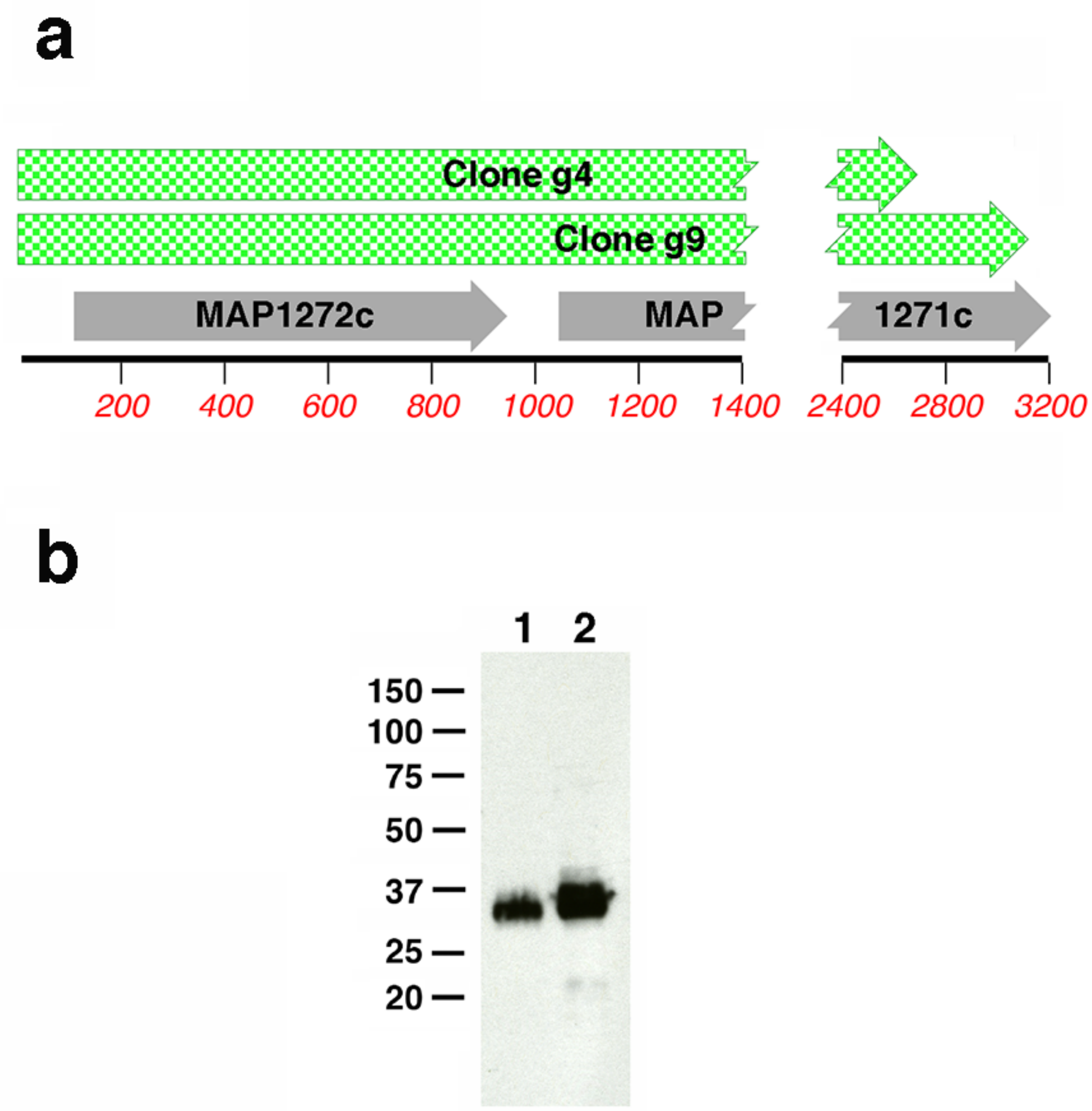

Supplemental Figure S5. MAP1272c is detected in vaccinated and infected goats. Sequence analysis of expression library subclone inserts shows two overlapping sequences aligned with the *Map* K-10 genome (**a**). Annotated K-10 genes are shown in gray. Clone insert sizes and genome coordinates are shown in Supplemental table S1. (**b**) Immunoblot analysis of IPTG-induced lysates harboring the clones in (**b**). Pooled goat sera diluted 1:400 served as the primary antibody. Kilodalton size standards are indicated in the left margin. Lanes: 1=Clone g4; 2=Clone g9. Note that only MAP1272c is common to both inserts in (**a**) and is the expected size. MAP1272c is 29 kDa and MAP1271c is 78 kDa.

Supplemental table S2. Relative antigenicity of recombinant proteins probed with two infected goats and two vaccinated goats.

		Infected goats						Vaccinated goats					
		Goat 13			Goat 21			Goat 56			Goat 67		
Spot address	Protein	Backgrd Subtracted Inegrated Density	Backgrd Subtracted MBP Spot Control	Relative antigenicity corrected by MBP loading	Backgrd Subtracted Inegrated Density	Backgrd Subtracted MBP Spot Control	Relative antigenicity corrected by MBP loading	Backgrd Subtracted Inegrated Density	Backgrd Subtracted MBP Spot Control	Relative antigenicity corrected by MBP loading	Backgrd Subtracted Inegrated Density	Backgrd Subtracted MBP Spot Control	Relative antigenicity corrected by MBP loading
3A1	MAP_2168c	51864	154284	0.33616	55893	154284	0.36227	66620	154284	0.43180	58243	154284	0.37751
3A2	MAP_2858	30044	133983	0.22424	32777	133983	0.24464	23199	133983	0.17315	33387	133983	0.24919
3A3	MAP_1885c	0	54796	0.00000	19206	54796	0.35050	4234	54796	0.07727	8803	54796	0.16065
3A4	MAP_3864	7120	179986	0.03956	30471	179986	0.16930	41335	179986	0.22966	10842	179986	0.06024
3A5	MAP_2864c dapA_2	97717	299749	0.32600	129737	299749	0.43282	104809	299749	0.34966	117022	299749	0.39040
3A6	MAP_1505	83168	185634	0.44802	129252	185634	0.69627	36609	185634	0.19721	38563	185634	0.20774
3A7	MAP_1682c	177551	236596	0.75044	201434	236596	0.85138	60446	236596	0.25548	108455	236596	0.45840
3A8	MAP_0810 cspB	22264	91416	0.24355	12372	91416	0.13534	37640	91416	0.41174	48568	91416	0.53129
3A9	MAP_4254	179137	242793	0.73782	122808	242793	0.50581	19846	242793	0.08174	71427	242793	0.29419
3A10	MAP_3737	217827	289688	0.75194	245998	289688	0.84918	55988	289688	0.19327	232683	289688	0.80322
3A11	MAP_3423c glpD2	330571	160980	2.05349	156812	160980	0.97411	51109	160980	0.31749	83934	160980	0.52139
3A12	MAP_1228 lipL	97711	163643	0.59710	52803	163643	0.32267	81918	163643	0.50059	45222	163643	0.27635
3B1	MAP_4006	84152	19813	4.24731	122034	19813	6.15929	11118	19813	0.56115	31386	19813	1.58411
3B2	MAP_2987c glnB	1599	102287	0.01563	23202	102287	0.22683	19362	102287	0.18929	63777	102287	0.62351
3B3	MAP_1234	101202	223915	0.45197	187258	223915	0.83629	72496	223915	0.32377	99596	223915	0.44479
3B4	MAP_3421c	64608	59089	1.09340	62352	59089	1.05522	22605	59089	0.38256	25800	59089	0.43663
3B5	MAP_3200	36807	237746	0.15482	133319	237746	0.56076	40840	237746	0.17178	47551	237746	0.20001
3B6	MAP_4106	49866	227231	0.21945	112611	227231	0.49558	288893	227231	1.27136	253047	227231	1.11361
3B7	MAP_0574 pstB	85619	156096	0.54850	112459	156096	0.72045	37575	156096	0.24072	49790	156096	0.31897
3B8	MAP_3889	30269	15642	1.93511	5264	15642	0.33653	5405	15642	0.34554	67825	15642	4.33608
3B9	MAP_0191c	208070	196216	1.06041	225310	196216	1.14828	92838	196216	0.47314	87872	196216	0.44783
3B10	MAP_4265 groEL1	48957	153799	0.31832	82218	153799	0.53458	40961	153799	0.26633	59669	153799	0.38797
3B11	MAP_0117	34967	166328	0.21023	34551	166328	0.20773	39704	166328	0.23871	43009	166328	0.25858
3B12	MAP_1110 adi	9387	187787	0.04999	88790	187787	0.47282	49902	187787	0.26574	38273	187787	0.20381
3C1	MAP_3227c	110419	68806	1.60479	67114	68806	0.97541	117538	68806	1.70825	46001	68806	0.66856
3C2	MAP_0170 sigI	32881	284120	0.11573	70139	284120	0.24686	70097	284120	0.24672	28692	284120	0.10099
3C3	MAP_3316 entC	41618	181070	0.22984	60042	181070	0.33160	79282	181070	0.43785	79346	181070	0.43821
3C4	MAP_0118	139290	209451	0.66502	161437	209451	0.77076	99494	209451	0.47502	134416	209451	0.64175
3C5	MAP_0887c	122030	275082	0.44361	89926	275082	0.32691	50799	275082	0.18467	62282	275082	0.22641
3C6	MAP_0578	101209	277735	0.36441	134819	277735	0.48542	19142	277735	0.06892	53485	277735	0.19258
3C7	MAP_0730c	137706	187362	0.73497	120376	187362	0.64248	48682	187362	0.25983	91972	187362	0.49088
3C8	MAP_1393c	157143	341448	0.46023	156007	341448	0.45690	27592	341448	0.08081	18958	341448	0.05552

Supplemental Table S2. Spot intensities from dot blots exposed to sera from infected goats 13 and 21 as well as vaccinated goats 56 and 67. Shown is the spot address along with the corresponding protein and background subtracted integrated density results. This result was divided by the background subtracted MBP loading control to obtain the relative antigenicity.

Supplemental table S2 (continued). Relative antigenicity of recombinant proteins probed with two infected goats and two vaccinated goats.

		Infected goats						Vaccinated goats					
		Goat 13			Goat 21			Goat 56			Goat 67		
Spot address	Protein	Backgrd Subtracted Inegrated Density	Backgrd Subtracted MBP Spot Control	Relative antigenicity corrected by MBP loading	Backgrd Subtracted Inegrated Density	Backgrd Subtracted MBP Spot Control	Relative antigenicity corrected by MBP loading	Backgrd Subtracted Inegrated Density	Backgrd Subtracted MBP Spot Control	Relative antigenicity corrected by MBP loading	Backgrd Subtracted Inegrated Density	Backgrd Subtracted MBP Spot Control	Relative antigenicity corrected by MBP loading
3C9	MAP_4186 rplO	55338	76212	0.72611	29540	76212	0.38760	80521	76212	1.05654	37124	76212	0.48711
3C10	MAP_3936 groEL2	362749	232885	1.55763	104209	232885	0.44747	65749	232885	0.28232	90315	232885	0.38781
3C11	MAP_0245c	51294	282857	0.18134	31774	282857	0.11233	34826	282857	0.12312	19063	282857	0.06739
3C12	MAP_1184c	47853	249101	0.19210	86901	249101	0.34886	22892	249101	0.09190	13163	249101	0.05284
3D1	MAP_1938c	179358	143269	1.25190	245381	143269	1.71273	63289	143269	0.44175	73896	143269	0.51578
3D2	MAP_1210 inhA	17672	192961	0.09158	32957	192961	0.17080	30319	192961	0.15713	42214	192961	0.21877
3D3	MAP_2456c atpE	54157	297586	0.18199	143766	297586	0.48311	53416	297586	0.17950	33153	297586	0.11141
3D4	MAP_0251c	4627	99085	0.04670	31181	99085	0.31469	7812	99085	0.07884	7762	99085	0.07834
3D5	MAP_2535	42833	51807	0.82678	59985	51807	1.15786	23074	51807	0.44538	13084	51807	0.25255
3D6	MAP_1998 kasA	188586	267514	0.70496	282647	267514	1.05657	60361	267514	0.22564	98807	267514	0.36935
3D7	MAP_1099	117718	59882	1.96583	215044	59882	3.59113	41825	59882	0.69846	84965	59882	1.41887
3D8	MAP_1662c	48953	124002	0.39478	72951	124002	0.58831	15003	124002	0.12099	12938	124002	0.10434
3D9	MAP_3968 hbha	228899	97313	2.35219	176951	97313	1.81837	57856	97313	0.59454	72680	97313	0.74687
3D10	MAP_3538	42889	249499	0.17190	103177	249499	0.41354	18769	249499	0.07523	9197	249499	0.03686
3D11	MAP_0506c	103698	196811	0.52689	160897	196811	0.81752	50700	196811	0.25761	33688	196811	0.17117
3D12	MAP_1238c drrA	128681	351587	0.36600	172667	351587	0.49111	38596	351587	0.10978	15626	351587	0.04444
3 E1	MAP_4141 rpsG	5107	54539	0.09364	0	54539	0.00000	381891	54539	7.00216	341505	54539	6.26167
3 E2	MAP_3720	111631	220747	0.50570	198969	220747	0.90134	79407	220747	0.35972	27696	220747	0.12546
3 E3	MAP_0540	51744	182529	0.28348	137140	182529	0.75133	16118	182529	0.08830	26580	182529	0.14562
3 E4	MAP_1056	43234	346610	0.12473	120929	346610	0.34889	18475	346610	0.05330	31071	346610	0.08964
3 E5	MAP_3788	85912	231434	0.37122	148718	231434	0.64259	41090	231434	0.17755	51257	231434	0.22148
3 E6	MAP_3555	276778	364796	0.75872	215465	364796	0.59065	52022	364796	0.14261	51733	364796	0.14181
3 E7	MAP_1075c	77337	199678	0.38731	201878	199678	1.01102	22778	199678	0.11407	19255	199678	0.09643
3 E8	MAP_2129	143824	212738	0.67606	241139	212738	1.13350	36284	212738	0.17056	88804	212738	0.41743
3 E9	MAP_4272c	211525	279861	0.75582	159334	279861	0.56933	25493	279861	0.09109	8226	279861	0.02939
3 E10	MAP_1834c prcA	210318	188689	1.11463	364784	188689	1.93326	59029	188689	0.31284	51602	188689	0.27348
3 E11	MAP_1001c	126369	190138	0.66462	170110	190138	0.89467	40751	190138	0.21432	24130	190138	0.12691
3 E12	MAP_3627	33773	121404	0.27819	55071	121404	0.45362	16700	121404	0.13756	8651	121404	0.07126
3F1	MPB83	38955	243405	0.16004	103519	243405	0.42530	24524	243405	0.10075	29899	243405	0.12284
3F2	MAP_3444	78115	288696	0.27058	264132	288696	0.91491	73909	288696	0.25601	55611	288696	0.19263
3F3	MAP_3577 fabG3_2	133948	190035	0.70486	227638	190035	1.19787	14350	190035	0.07551	10235	190035	0.05386
3F4	MAP_0966c	233200	249121	0.93609	278913	249121	1.11959	86682	249121	0.34795	108991	249121	0.43750

Supplemental Table S2 (continued). Spot intensities from dot blots exposed to sera from infected goats 13 and 21 as well as vaccinated goats 56 and 67. Shown is the spot address along with the corresponding protein and background subtracted integrated density results. This result was divided by the background subtracted MBP loading control to obtain the relative antigenicity.

Supplemental table S2 (continued). Relative antigenicity of recombinant proteins probed with two infected goats and two vaccinated goats.

		Infected goats						Vaccinated goats					
		Goat 13			Goat 21			Goat 56			Goat 67		
Spot address	Protein	Backgrd Subtracted Inegrated Density	Backgrd Subtracted MBP Spot Control	Relative antigenicity corrected by MBP loading	Backgrd Subtracted Inegrated Density	Backgrd Subtracted MBP Spot Control	Relative antigenicity corrected by MBP loading	Backgrd Subtracted Inegrated Density	Backgrd Subtracted MBP Spot Control	Relative antigenicity corrected by MBP loading	Backgrd Subtracted Inegrated Density	Backgrd Subtracted MBP Spot Control	Relative antigenicity corrected by MBP loading
3F5	MAP_0284c	169151	326811	0.51758	186504	326811	0.57068	46967	326811	0.14371	23341	326811	0.07142
3F6	MAP_3190 fadB4	55131	235149	0.23445	63666	235149	0.27075	12918	235149	0.05494	33889	235149	0.14412
3F7	MAP_2720c	58784	217220	0.27062	125794	217220	0.57911	50383	217220	0.23194	42651	217220	0.19635
3F8	MAP_3290c mpt64	111103	307496	0.36132	183938	307496	0.59818	18073	307496	0.05877	23082	307496	0.07506
3F9	MAP_0833c	143934	232807	0.61825	262232	232807	1.12639	39461	232807	0.16950	78089	232807	0.33542
3F10	MAP_0593c	71905	232667	0.30905	83933	232667	0.36074	23904	232667	0.10274	11542	232667	0.04961
3F11	MAP_2646c fadA6_3	90517	87276	1.03714	114401	87276	1.31080	26427	87276	0.30280	24151	87276	0.27672
3F12	MBP-lacZ (Control)	32686	109356	0.29890	62234	109356	0.56910	3749	109356	0.03428	4789	109356	0.04379
3G1	MAP_1298 impA	57055	254782	0.22394	103746	254782	0.40720	33738	254782	0.13242	22350	254782	0.08772
3G2	MAP_4229	105160	176807	0.59477	219485	176807	1.24138	47728	176807	0.26994	45900	176807	0.25961
3G3	MAP_3393c purE	18640	185151	0.10067	38908	185151	0.21014	12510	185151	0.06757	16675	185151	0.09006
3G4	MAP_0435c ppa	30145	115573	0.26083	34704	115573	0.30028	13109	115573	0.11343	27361	115573	0.23674
3G5	MAP_3679	123638	184276	0.67094	223017	184276	1.21023	14077	184276	0.07639	13193	184276	0.07159
3G6	MAP_1588c ahpD	24962	255177	0.09782	27649	255177	0.10835	9043	255177	0.03544	6917	255177	0.02711
3G7	MAP_4232 rpsD	27316	12594	2.16897	74945	12594	5.95085	101332	12594	8.04605	70275	12594	5.58004
3G8	MAP_4287c	16551	139809	0.11838	31058	139809	0.22215	296	139809	0.00212	9928	139809	0.07101
3G9	MAP_0782	56217	192938	0.29137	86143	192938	0.44648	22669	192938	0.11749	7241	192938	0.03753
3G10	MAP_1653 tpx	17047	65150	0.26166	68873	65150	1.05715	10895	65150	0.16723	12277	65150	0.18844
3G11	MAP_2312c fadE19	88267	389664	0.22652	203416	389664	0.52203	41810	389664	0.10730	26182	389664	0.06719
3G12	MAP_0440c lpqG	28004	267749	0.10459	92910	267749	0.34700	11852	267749	0.04427	8493	267749	0.03172
3H1	MAP_3024c hupB	92101	73456	1.25383	154043	73456	2.09708	11454	73456	0.15593	20292	73456	0.27625
3H2	MAP_3061c fixA	63639	161764	0.39341	86763	161764	0.53636	67481	161764	0.41716	90079	161764	0.55685
3H3	MAP_3701c hsp	26737	117233	0.22807	78995	117233	0.67383	8581	117233	0.07320	10687	117233	0.09116
3H4	MAP_2705c	5021	130479	0.03848	23470	130479	0.17988	4994	130479	0.03827	13796	130479	0.10573
3H5	MAP_3073	45611	78000	0.58476	81232	78000	1.04144	37020	78000	0.47462	15323	78000	0.19645
3H6	MAP_0516c echA20	158363	348138	0.45489	254400	348138	0.73074	30490	348138	0.08758	17880	348138	0.05136
3H7	MAP_0242c	24837	148680	0.16705	65122	148680	0.43800	10276	148680	0.06911	18277	148680	0.12293
3H8	MAP_2128c	95275	257252	0.37036	163824	257252	0.63682	9854	257252	0.03830	47289	257252	0.18382
3H9	MAP_0036	129019	116079	1.11148	165185	116079	1.42304	54466	116079	0.46921	47326	116079	0.40771
3H10	MAP_2280c clpP2	38881	147470	0.26365	81361	147470	0.55171	7440	147470	0.05045	32871	147470	0.22290
3H11	MAP_1101	61147	257306	0.23764	86653	257306	0.33677	11853	257306	0.04607	12172	257306	0.04731
3H12	Sonicate	295712	1820	162.47912	379766	1820	208.66264	113621	1820	62.42912	417164	1820	229.21099

Supplemental Table S2 (continued). Spot intensities from dot blots exposed to sera from infected goats 13 and 21 as well as vaccinated goats 56 and 67. Shown is the spot address along with the corresponding protein and background subtracted integrated density results. This result was divided by the background subtracted MBP loading control to obtain the relative antigenicity.

Supplemental table S2 (continued). Relative antigenicity of recombinant proteins probed with two infected goats and two vaccinated goats.

		Infected goats						Vaccinated goats					
		Goat 13			Goat 21			Goat 56			Goat 67		
Spot address	Protein	Backgrd Subtracted Inegrated Density	Backgrd Subtracted MBP Spot Control	Relative antigenicity corrected by MBP loading	Backgrd Subtracted Inegrated Density	Backgrd Subtracted MBP Spot Control	Relative antigenicity corrected by MBP loading	Backgrd Subtracted Inegrated Density	Backgrd Subtracted MBP Spot Control	Relative antigenicity corrected by MBP loading	Backgrd Subtracted Inegrated Density	Backgrd Subtracted MBP Spot Control	Relative antigenicity corrected by MBP loading
4A1	MAP_3176 fprA	36336	174913	0.20774	5259	174913	0.03007	15235	174913	0.08710	49366	174913	0.28223
4A2	MAP_2795	220220	276674	0.79595	161660	276674	0.58430	336825	276674	1.21741	264752	276674	0.95691
4A3	MAP_1420 (F3-B2)	37154	221776	0.16753	33237	221776	0.14987	39527	221776	0.17823	90861	221776	0.40970
4A4	MAP_2344	16709	112761	0.14818	0	112761	0.00000	14948	112761	0.13256	8405	112761	0.07454
4A5	MAP_0875c	137071	238995	0.57353	119904	238995	0.50170	52272	238995	0.21872	81204	238995	0.33977
4A6	MAP_1933	179027	215752	0.82978	183345	215752	0.84980	168194	215752	0.77957	129698	215752	0.60114
4A7	MAP_1742c	90231	262007	0.34438	68936	262007	0.26311	44731	262007	0.17072	72947	262007	0.27842
4A8	MAP_1261 plsB1	171251	274415	0.62406	115054	274415	0.41927	54906	274415	0.20008	50575	274415	0.18430
4A9	MAP_0997c kdpC	111027	246031	0.45127	119094	246031	0.48406	72180	246031	0.29338	112400	246031	0.45685
4A10	MAP_1142	58890	228537	0.25768	66925	228537	0.29284	61368	228537	0.26853	37741	228537	0.16514
4A11	MAP_0522	52906	250667	0.21106	56702	250667	0.22620	49769	250667	0.19855	33425	250667	0.13334
4A12	MAP_0178c	153221	185664	0.82526	73514	185664	0.39595	35134	185664	0.18923	30983	185664	0.16688
4B1	MAP_3668c	184840	252284	0.73267	105038	252284	0.41635	171207	252284	0.67863	76468	252284	0.30310
4B2	MAP_2944c cdsA	253213	207498	1.22032	221961	207498	1.06970	214250	207498	1.03254	224889	207498	1.08381
4B3	MAP_2212c cysT	164813	270823	0.60856	119483	270823	0.44118	104735	270823	0.38673	166584	270823	0.61510
4B4	MAP_2299c	131543	142868	0.92073	163938	142868	1.14748	75259	142868	0.52677	131225	142868	0.91851
4B5	MAP_0854 ndh	302433	147020	2.05709	349618	147020	2.37803	228753	147020	1.55593	209184	147020	1.42283
4B6	MAP_1567	138604	96309	1.43916	130265	96309	1.35257	57135	96309	0.59325	43493	96309	0.45160
4B7	MAP_1934 qcrA	51218	142830	0.35859	59860	142830	0.41910	34825	142830	0.24382	15155	142830	0.10611
4B8	MAP_1418c	98991	152321	0.64988	91081	152321	0.59795	30724	152321	0.20171	32505	152321	0.21340
4B9	MAP_1137c	99958	175325	0.57013	77192	175325	0.44028	-5229	175325	-0.02982	19706	175325	0.11240
4B10	MAP_0752c	44814	151922	0.29498	40312	151922	0.26535	54002	151922	0.35546	24730	151922	0.16278
4B11	MAP_0078	10347	32097	0.32237	14102	32097	0.43936	31915	32097	0.99433	4617	32097	0.14385
4B12	MAP_0411 dppC	304081	144356	2.10647	345585	144356	2.39398	33640	144356	0.23303	83784	144356	0.58040
4C1	MAP_1025 pra	63084	253057	0.24929	33718	253057	0.13324	76784	253057	0.30343	39903	253057	0.15768
4C2	MAP_2808 trkA	50678	210988	0.24019	45981	210988	0.21793	73035	210988	0.34616	23123	210988	0.10959
4C3	MAP_1799c	133485	362512	0.36822	118584	362512	0.32712	53216	362512	0.14680	108423	362512	0.29909
4C4	MAP_2289c	85559	292725	0.29228	94882	292725	0.32413	87300	292725	0.29823	50423	292725	0.17225
4C5	MAP_2093c	12470	67498	0.18475	18434	67498	0.27310	13221	67498	0.19587	2072	67498	0.03070
4C6	MAP_1782c	52489	193931	0.27066	44267	193931	0.22826	33042	193931	0.17038	21717	193931	0.11198
4C7	MAP_1546c	56667	261199	0.21695	33028	261199	0.12645	68822	261199	0.26348	14964	261199	0.05729
4C8	MAP_1301 chaA	167938	206518	0.81319	155787	206518	0.75435	49592	206518	0.24013	53609	206518	0.25959

Supplemental Table S2 (continued). Spot intensities from dot blots exposed to sera from infected goats 13 and 21 as well as vaccinated goats 56 and 67. Shown is the spot address along with the corresponding protein and background subtracted integrated density results. This result was divided by the background subtracted MBP loading control to obtain the relative antigenicity.

Supplemental table S2 (continued). Relative antigenicity of recombinant proteins probed with two infected goats and two vaccinated goats.

		Infected goats						Vaccinated goats					
		Goat 13			Goat 21			Goat 56			Goat 67		
Spot address	Protein	Backgrd Subtracted Inegrated Density	Backgrd Subtracted MBP Spot Control	Relative antigenicity corrected by MBP loading	Backgrd Subtracted Inegrated Density	Backgrd Subtracted MBP Spot Control	Relative antigenicity corrected by MBP loading	Backgrd Subtracted Inegrated Density	Backgrd Subtracted MBP Spot Control	Relative antigenicity corrected by MBP loading	Backgrd Subtracted Inegrated Density	Backgrd Subtracted MBP Spot Control	Relative antigenicity corrected by MBP loading
4C9	MAP_0727	84988	224794	0.37807	68578	224794	0.30507	28304	224794	0.12591	28643	224794	0.12742
4C10	MAP_0690	57156	179928	0.31766	47075	179928	0.26163	71782	179928	0.39895	19386	179928	0.10774
4C11	MAP_0613c	117185	247823	0.47286	150440	247823	0.60705	68526	247823	0.27651	30942	247823	0.12486
4C12	MAP_0257	134762	211056	0.63851	205445	211056	0.97341	50019	211056	0.23699	75402	211056	0.35726
4D1	MAP_3089c	44847	279338	0.16055	69654	279338	0.24935	93868	279338	0.33604	61792	279338	0.22121
4D2	MAP_3527 pepA	368051	139916	2.63051	377795	139916	2.70016	290076	139916	2.07322	378256	139916	2.70345
4D3	MAP_1593	142224	373706	0.38058	27440	373706	0.07343	38621	373706	0.10335	26366	373706	0.07055
4D4	MAP_2234	154181	155462	0.99176	242658	155462	1.56088	54786	155462	0.35241	174655	155462	1.12346
4D5	MAP_1808c	91438	190010	0.48123	130271	190010	0.68560	84242	190010	0.44336	63725	190010	0.33538
4D6	MAP_4088	70920	45740	1.55050	93594	45740	2.04622	102178	45740	2.23389	130835	45740	2.86041
4D7	MAP_1866c	11139	92923	0.11987	0	92923	0.00000	22508	92923	0.24222	4508	92923	0.04851
4D8	MAP_1315c cydB	93740	99880	0.93853	86170	99880	0.86274	22887	99880	0.22914	27429	99880	0.27462
4D9	MAP_1236c drrC	227715	140092	1.62547	208877	140092	1.49100	126578	140092	0.90353	141279	140092	1.00847
4D10	MAP_0473c radA	31597	118459	0.26673	45953	118459	0.38792	65352	118459	0.55168	21251	118459	0.17940
4D11	MAP_0405c	137011	286489	0.47824	166599	286489	0.58152	43357	286489	0.15134	29997	286489	0.10471
4D12	MAP_0051c gluA	67405	176112	0.38274	78835	176112	0.44764	21459	176112	0.12185	32193	176112	0.18280
4 E1	MAP_3567	115938	320487	0.36176	130646	320487	0.40765	115134	320487	0.35925	192865	320487	0.60179
4 E2	MAP_1050c ppiB	300322	219339	1.36921	259108	219339	1.18131	329572	219339	1.50257	262883	219339	1.19852
4 E3	MAP_1575c	332835	250607	1.32812	283862	250607	1.13270	249363	250607	0.99504	206383	250607	0.82353
4 E4	MAP_2211c	158620	193167	0.82115	129060	193167	0.66813	134273	193167	0.69511	133142	193167	0.68926
4 E5	MAP_1932 ctaE	36157	91504	0.39514	50216	91504	0.54878	49500	91504	0.54096	17749	91504	0.19397
4 E6	MAP_1113c	19830	73975	0.26806	0	73975	0.00000	50895	73975	0.68800	38545	73975	0.52105
4 E7	MAP_1496c	57340	192818	0.29738	48674	192818	0.25243	25946	192818	0.13456	40193	192818	0.20845
4 E8	MAP_1273c	244014	109218	2.23419	269366	109218	2.46632	78183	109218	0.71584	129830	109218	1.18872
4 E9	MAP_0619c	55466	167637	0.33087	56142	167637	0.33490	37683	167637	0.22479	16546	167637	0.09870
4 E10	MAP_0612	77625	205836	0.37712	70581	205836	0.34290	85230	205836	0.41407	75345	205836	0.36604
4 E11	MAP_0618c	133984	229984	0.58258	107388	229984	0.46694	54361	229984	0.23637	28935	229984	0.12581
4 E12	MAP_0009	34290	165510	0.20718	33531	165510	0.20259	20249	165510	0.12234	22576	165510	0.13640
4F1	MAP_3185	161776	243211	0.66517	149344	243211	0.61405	260414	243211	1.07073	237493	243211	0.97649
4F2	MAP_3610	258013	344707	0.74850	264646	344707	0.76774	146439	344707	0.42482	117362	344707	0.34047
4F3	MAP_2457c atpD	201667	258912	0.77890	218599	258912	0.84430	186344	258912	0.71972	96326	258912	0.37204
4F4	MAP_2210c	192874	194148	0.99344	266512	194148	1.37273	387124	194148	1.99396	278730	194148	1.43566

Supplemental Table S2 (continued). Spot intensities from dot blots exposed to sera from infected goats 13 and 21 as well as vaccinated goats 56 and 67. Shown is the spot address along with the corresponding protein and background subtracted integrated density results. This result was divided by the background subtracted MBP loading control to obtain the relative antigenicity.

Supplemental table S2 (continued). Relative antigenicity of recombinant proteins probed with two infected goats and two vaccinated goats.

		Infected goats						Vaccinated goats					
		Goat 13			Goat 21			Goat 56			Goat 67		
Spot address	Protein	Backgrd Subtracted Ingrated Density	Backgrd Subtracted MBP Spot Control	Relative antigenicity corrected by MBP loading	Backgrd Subtracted Inegrated Density	Backgrd Subtracted MBP Spot Control	Relative antigenicity corrected by MBP loading	Backgrd Subtracted Inegrated Density	Backgrd Subtracted MBP Spot Control	Relative antigenicity corrected by MBP loading	Backgrd Subtracted Inegrated Density	Backgrd Subtracted MBP Spot Control	Relative antigenicity corrected by MBP loading
4F5	MAP_1535 pgsA2	95770	238680	0.40125	166368	238680	0.69703	113782	238680	0.47671	101735	238680	0.42624
4F6	MAP_1907c	72917	218796	0.33326	121443	218796	0.55505	59748	218796	0.27308	59453	218796	0.27173
4F7	MAP_1410	48600	277724	0.17499	62707	277724	0.22579	43982	277724	0.15837	66474	277724	0.23935
4F8	MAP_1229	109904	213440	0.51492	133177	213440	0.62396	53159	213440	0.24906	40462	213440	0.18957
4F9	MAP_0704	87404	290399	0.30098	102153	290399	0.35177	83522	290399	0.28761	81169	290399	0.27951
4F10	MAP_0773	94350	264055	0.35731	109759	264055	0.41567	81366	264055	0.30814	23422	264055	0.08870
4F11	MAP_0600c	143225	287784	0.49768	238013	287784	0.82705	68959	287784	0.23962	54476	287784	0.18929
4F12	MBP-lacZ (Control)	15533	222558	0.06979	3256	222558	0.01463	9552	222558	0.04292	2565	222558	0.01153
4G1	MAP_2809 trkB	36461	216470	0.16843	30238	216470	0.13969	29058	216470	0.13424	58562	216470	0.27053
4G2	MAP_0872 phoS2_2	177799	200693	0.88593	203869	200693	1.01583	145611	200693	0.72554	207036	200693	1.03161
4G3	MAP_2477c	71090	242949	0.29261	40196	242949	0.16545	39788	242949	0.16377	137298	242949	0.56513
4G4	MAP_2175c	116780	284106	0.41104	59101	284106	0.20802	131198	284106	0.46179	63969	284106	0.22516
4G5	MAP_1740c	42631	256530	0.16618	53369	256530	0.20804	35493	256530	0.13836	63798	256530	0.24870
4G6	MAP_1356c	103861	263264	0.39451	78517	263264	0.29824	43765	263264	0.16624	29211	263264	0.11096
4G7	MAP_1423	121175	159031	0.76196	156745	159031	0.98563	51926	159031	0.32651	74822	159031	0.47049
4G8	MAP_0959	47369	155400	0.30482	62310	155400	0.40097	37929	155400	0.24407	27212	155400	0.17511
4G9	MAP_0668	23350	271211	0.08610	83137	271211	0.30654	46910	271211	0.17296	29164	271211	0.10753
4G10	MAP_0818c	110662	384171	0.28805	156009	384171	0.40609	80327	384171	0.20909	34616	384171	0.09011
4G11	MAP_0410 dppB	5423	57519	0.09428	91926	57519	1.59818	21837	57519	0.37965	24332	57519	0.42303
4G12	MAP_0026 fadD33_1	6737	160808	0.04189	50370	160808	0.31323	9706	160808	0.06036	27262	160808	0.16953
4H1	MAP_2875	128643	170797	0.75319	112122	170797	0.65646	87640	170797	0.51312	45753	170797	0.26788
4H2	MAP_1775	204045	114253	1.78590	162892	114253	1.42571	169372	114253	1.48243	242315	114253	2.12086
4H3	MAP_2356	99686	343409	0.29028	51455	343409	0.14984	92667	343409	0.26984	53529	343409	0.15588
4H4	MAP_0998c	101552	286921	0.35394	86212	286921	0.30047	155991	286921	0.54367	203540	286921	0.70939
4H5	MAP_1818c	137590	122600	1.12227	154480	122600	1.26003	118478	122600	0.96638	173702	122600	1.41682
4H6	MAP_1433c	188140	286000	0.65783	221816	286000	0.77558	99425	286000	0.34764	190301	286000	0.66539
4H7	MAP_1394c amt_1	34332	196298	0.17490	47803	196298	0.24352	103631	196298	0.52793	29077	196298	0.14813
4H8	MAP_0687	172051	251296	0.68465	144360	251296	0.57446	83909	251296	0.33391	101275	251296	0.40301
4H9	MAP_0691c	20928	137094	0.15265	39139	137094	0.28549	18730	137094	0.13662	28377	137094	0.20699
4H10	MAP_0714c	64495	330459	0.19517	176249	330459	0.53335	52757	330459	0.15965	38061	330459	0.11518
4H11	MAP_0547	3801	364253	0.01044	84930	364253	0.23316	31062	364253	0.08528	36005	364253	0.09885
4H12	Sonicate	26019	17873	1.45577	380951	17873	21.31433	62676	17873	3.50674	422749	17873	23.65294

Supplemental Table S2 (continued). Spot intensities from dot blots exposed to sera from infected goats 13 and 21 as well as vaccinated goats 56 and 67. Shown is the spot address along with the corresponding protein and background subtracted integrated density results. This result was divided by the background subtracted MBP loading control to obtain the relative antigenicity.
